# NonO Is a Novel Co-factor of PRDM1 and Regulates Inflammatory Response in Monocyte Derived-Dendritic Cells

**DOI:** 10.3389/fimmu.2020.01436

**Published:** 2020-07-10

**Authors:** Kyungwoo Lee, Su Hwa Jang, Hong Tian, Sun Jung Kim

**Affiliations:** ^1^Institute of Molecular Medicine, The Feinstein Institute for Medical Research, Manhasset, NY, United States; ^2^Department of Biomedical Science, Graduate School of Biomedical Sciences and Engineering, Hanyang University, Seoul, South Korea

**Keywords:** PRDM1, NonO, IL-6, inflammation, dendritic cells

## Abstract

Proper expression of the transcription factor, Positive regulatory domain 1 (*PRDM1*), is required for maintaining homeostasis of human monocyte derived-dendritic cells (MO-DCs). The molecular mechanisms and gene targets of PRDM1 in B and T lymphocytes have been identified. However, the function of PRDM1 in dendritic cells (DCs) remains unclear. We investigate co-regulators of PRDM1 in MO-DCs identified by mass spectrometry (MS) and co-immunoprecipitation (Co-IP). Notably, non-POU domain-containing octamer-binding protein (NonO) was found to be a PRDM1 binding protein in the nucleus of MO-DCs. NonO is recruited to the PRDM1 binding site in the promoter region of IL-6. Knockdown of NonO expression by siRNA lessened suppression of IL-6 promoter activity by PRMD1 following LPS stimulation. While NonO binding to PRDM1 was observed in human myeloma cell lines, an effect of NonO on IL-6 expression was not observed. Thus, loss of NonO interrupted the inhibitory effect of PRDM1 on IL-6 expression in MO-DCs, but not plasma cells. Moreover, MO-DCs with low expression of PRDM1 or NonO induce an increased number of IL-21-producing T_FH_-like cells *in vitro*. These data suggest that low level of PRDM1 and NonO lead to enhanced activation of MO-DCs and the regulation of MO-DC function by PRDM1 is mediated through cell lineage-specific mechanisms.

## Introduction

Positive regulatory domain 1 (PRDM1, also named BLIMP1) was identified as a repressor of interferon beta (IFN-β) gene expression in humans and mice (1, 2). PRDM1 is expressed in multiple cell lineages and is critical for early development (2–4). The immunological function of PRDM1 was first identified in B lymphocytes. Expression of PRDM1 is strongly induced in post-germinal center B cells committed to plasma cell (PC) differentiation (5, 6). In PCs, PRDM1 acts as a master transcription factor through positive regulation of genes involved in plasmablast (PB) and PC function, and the absence of PRDM1 in B cells in mice leads to a lack of PC with hypoimmunoglobulinemia despite normal B cell memory responses (7–9).

Genome-wide association studies (GWAS) have identified polymorphisms in *PRDM1* that are associated with autoimmune diseases. Single nucleotide polymorphisms (SNPs) predisposing to systemic lupus erythematosus (SLE) and rheumatoid arthritis (RA) are located in the intergenic region between *PRDM1* and *ATG5* (10). Monocyte derived-dendritic cells (MO-DCs), but not B cells derived from healthy female individuals with the rs548234 SNP, which is a risk factor for SLE, show a lower level of *PRDM1* expression, suggesting that a proper expression of PRDM1 in dendritic cells (DCs) is required for immunological homeostasis in a gender-specific manner (11).

Immunoregulatory functions of PRDM1 in myeloid cells have been reported; mice with a DC-specific knockout of *Prdm1* (*Prdm1* CKO) spontaneously develop a lupus-like phenotype (11). Increased expression of the proinflammatory cytokine Interleukin-6 (IL-6) in DCs of *Prdm1* CKO mice, following Toll-like receptor (TLR) 4 stimulation, leads to an enhanced differentiation of follicular helper T cells (T_FH_), revealing a potential pathogenic mechanism for *PRDM1* in autoimmune diseases (11). PRDM1 also participates in the process of antigen processing and presentation, and regulates expression of class II trans-activator (CIITA) in PCs and lymphocytes (12, 13), and cathepsin S (CTSS) in DCs (14). CTSS was higher in PRDM1-deficient DCs than in control DCs and increased CTSS activity contributes to development of autoantibodies and enhanced induction of T_FH_ cells in female *Pdrm1* CKO mice (14). In addition, PRDM1 was identified as a critical downstream regulator of the aryl hydrocarbon receptor (AHR) during MO-DC differentiation; a lack of AHR expression enhances monocytes to macrophages differentiation (15). These studies together suggest that PRDM1 mediates different regulatory functions in myeloid cells.

Studies in cell lines suggest that recruitment of chromatin regulators is important for the suppressive function of PRDM1 (16–19). Studies performed in primary lymphocytes showed that PRDM1 recruits cell-type specific co-factors in CD4+ T cells, CD8+ T cells, and in plasmablasts (20–22). While there are some common target genes among lymphocytes, the majority is cell type-dependent. These observations suggest that co-factors of PRDM1 are one of important contributor to cell-type dependent regulatory mechanisms of PRDM1. In this study, we identified co-factors of PRDM1 in MO-DCs by immunoprecipitation and mass spectrometry (IP-MS). Among the candidate proteins, a non-POU domain-containing octamer-binding protein (NonO, also named p54nrb) helps PRDM1 to suppress IL-6 expression by direct binding to the *IL6* promoter. Moreover, a deficiency of PRDM1 or NonO in MO-DCs increases differentiation of IL-21 producing T_FH_-like cells. Together, these observations suggest that PRDM1 and NonO together regulate DC activation.

## Materials and Methods

### Preparations of Peripheral Blood Mononuclear Cells (PBMCs) and MO-DCs Differentiation

The protocol for study of human blood was approved by the Institutional Review Board (approval number: 17-0075). PBMCs were purified from leukopack (NY Blood center) as described previously (14). To prepare MO-DCs, CD14+ monocytes were isolated from MO-DCs by EasySep Human CD14 positive selection kit II (StemCell Technologies) according to the manufacturer's protocol. CD14+ monocytes were cultured with RPMI1640 supplemented with 10% heat-inactivated fetal bovine serum (FBS), 1% penicillin-streptomycin (P/S), 1% L-glutamine, 100 ng/ml of recombinant human granulocyte-macrophage colony-stimulating factor (GM-CSF) (PeproTech), and 50 ng/ml of recombinant human IL-4 (PeproTech) for 7 days. Cultures were kept at 37°C in a humidified atmosphere and 5% CO_2_. On day 7, MO-DCs were collected only from the non-adherent cells and the purity of MO-DCs was confirmed by flow cytometry with antibodies which were purchased from eBioscience (anti-HLA-DR-FITC: LN3 and anti-CD209-PE/Cy7: eB-h209) (23). Over 85% of HLA-DR+CD209+ MO-DCs were obtained consistently. We excluded adherent cells since cells shows mixed population with CD209+ and CD209- with various degrees (Figure S1A).

### Cell Lines

The HEK-293 cell line was purchased from ATCC (ATCC CRL-1573) and maintained in DMEM with 10% FBS, 1% P/S, and 1% L-glutamine. The human myeloma cell lines U-266, RPMI-8266 and Daudi (a gift from Dr. Chiorazzi, FIMR, NY) were maintained in a 5% CO_2_ atmosphere in RPMI 1640 supplemented with 15% FBS, 1% P/S, and 1% L-glutamine.

### Co-IP and Mass Spectroscopy (MS) Assays

Co-IP was performed as described previously (24). Briefly, 2–5 μg of PRDM1 rabbit mAb (Cat# OAR03181, Aviva Systems Biology or cat# 9115s, Cell Signaling Technology) or normal rabbit immunoglobulin G (IgG) (Cat# 2729, Cell Signaling Technologies) were coupled to protein G or A-magnetic beads (DynaBead, Thermo Scientific). Anti-flag M2 magnetic beads (Milipore) were used to pull-down flag tagged RPDM1 in some experiments. Nuclear protein was extracted from PRDM1 transfected MO-DCs with a NE-PER nuclear and cytoplasmic extraction reagents kit (Thermo Scientific) and incubated with a bead-conjugated PRDM1 antibody or control IgG overnight at 4°C. The beads were washed and proteins bound by antibody were eluted by elution buffer and stored at −80°C until used for either immunoblotting or mass spectrometry. Mass spectrometry was performed, and analyses were done at cold spring harbor laboratory shared resources as described previously (25).

### Immunoblotting

Western blot was performed as described (24). Cell extracts or eluted proteins were separated by 4–12% Bis-Tris polyacrylamide gel electrophoresis (PAGE) (Invitrogen). Proteins were transferred to polyvinyliden difluoride (PVDF) membrane (GE Amersham, Hybond-C or Millipore, Immobilon-FL) and blocked for 1 h at room temperature with 5% non-fat dry milk in TBS-T buffer (20 mM Tris, 150 mM NaCl, 0.1% Tween20, pH 7.4). The membranes were then incubated with primary antibodies to HDAC1 (Cat# 5356s, Cell Signaling Technology), HDAC2 (Cat# 5113s, Cell signaling Technology), PRDM1 (Cat# 9115s), hnRNPM (Cat# SAB1404107, Sigma Aldrich), TP53BP1 (Cat# 4937s, Cell Signaling Technology), β-Actin (Cat# ab8226, Abcam), V5 (Cat# MA5-15253, Sigma Aldrich), and Flag (Cat# F1804, Sigma Aldrich) overnight at 4°C. Proteins bound by antibody were visualized by ECL (Thermo Scientific, # 34580 or Advansta, K-12045) and sapphire biomolecular imager (Azure Biosystems).

### Plasmids and Transient Transfections

Human *PRDM1* Tagged ORF Clone *PRDM1* (RC217363L1V) and human small interfering Ribonuclic acid (siRNA) oligo duplex exogenous (SR300437; *PRDM1* and SR321120; *NonO*) were purchased from Origene. *FLAG-NonO* (pCMV-myc-Flag-p54), *FLAG-TP53BP1* (pcDNA5-FRT/T0-Flag-53BP1), and *V5-hnRNPM* (pT7-V5-SBP-C1-HshnRNPM) expressing plasmids were purchased from Addgene. Transfections were prepared as described in previous study (24). For transient transfection to HEK-293 cells, 1–2 μg of plasmid was transfected to 70% confluent monolayered HEK-293 cells by Lipofectamin (Invitrogen). After 24 h, medium was replaced with complete medium and cells were further cultured for 2 days. 200 nM siRNA or 1–2 μg of plasmid was transfected to 10^6^ MO-DCs at day 5 during differentiation by Human Dendritic Cell Nucleofector™ Kits (Lonza). After transfection, MO-DCs were further differentiated for 2 days and cells were harvested for experiments. 10^6^ myeloma cells were transfected by using Nucleofector™ Kits (R kit for U-266, T kit for RPMI-8226, and V kit for Daudi) with 400 nM siRNA (Origene), according to manufacturer's instructions.

### Proximity Ligation Assay (PLA) Assay

The *in situ* PLA was performed on fixed MO-DCs with Duolink *in situ* Detection Reagents Red (Sigma Aldrich) according to the manufacturer's instructions. Briefly, cells were fixed with 4% paraformaldehyde (PFA) at room temperature and washed with PBS. Samples were permeabilized with 0.5% Triton-X-100 in PBS and blocked by blocking solution (provided by the kit) for 1 h at 37°C. Primary antibodies against NonO (Cat# sc-376865, Santa Cruz), hnRNPM, TP53BP1, PRDM1, V5, Flag or normal rabbit IgG (Cat# 2729, Cell Signaling Technology) were incubated overnight at 4°C. The samples were washed twice for 5 min with buffer A (provided by the kit), followed by incubation with the PLA probes (Sigma Aldrich) for 60 min at 37°C. Subsequent ligation for 30 min at 37°C and amplification for 100 min at 37°C were performed. Finally, the samples were mounted using Duolink *in situ* Mounting Medium with DAPI (Sigma Aldrich). Z-Stacks Images were captured using a 60X oil objective on Zeiss Apotome 2 microscope and LSM 880 confocal microscopy (Carl Zeiss Microscopy). Three-dimensional foci counting analysis was performed with Imaris software (Imaris v8.0.2).

### Chromatin Immunoprecipitation (ChIP) and PCR

ChIP assays were performed as previously described (14). 5 × 10^6^ MO-DCs were cross-linked with 1% formaldehyde for 10 min at room temperature and quenched with 125 mM glycine. Cells were washed with ice-cold 1X DPBS twice. Cell pellets were lysed in 300 μl ChIP Lysis Buffer I (50 mM HEPES.KOH, pH 7.5, 140 mM NaCl, 1 mM EDTA, pH 8.0, 10% Glycerol, 0.5% NP-40, 0.25% Triton X-100), ChIP Lysis Buffer II (10 mM Tris-HCl, pH 8.0, 200 mM NaCl, 1 mM EDTA, pH 8.0, 0.5 mM EGTA, pH 8.0), then ChIP Lysis Buffer III (10 mM Tris-HCl, pH 8.0, 100 mM NaCl, 1 mM EDTA, 0.5 mM EGTA, 0.1% sodium deoxycholate, 0.5% *N*-lauroylsarcosine). All three lysis buffers were supplemented with complete proteinase inhibitor (Roche), and each lysis was performed for 10 min at 4°C with rotation. After lysis, chromatin was sheared by sonication (7 cycles of 30 s ON and 60 s OFF by Q500 sonicator) (Fisher), which generated fragments ranging from 200 to 800 bp. Ten percent Triton X-100 was added to sonicated chromatin (nuclear membrane and lipids were removed by centrifuge). Ten percent of sonicated chromatin supernatant was saved as input control. Sonicated chromatin was incubated with 2 μg of antibody-Protein G and A Dynabeads (Invitrogen) complex overnight at 4°C. Unbound chromatin was removed with RIPA Buffer (50 mM HEPES.KOH, pH 7.5, 100 mM LiCl, 1 mM EDTA, 1% NP-40, 0.7% Sodium Deoxycholate), followed by one time washing with 10 mM pH 8.0 Tris elution buffer. Chromatin elution was done by incubation with elution buffer (50 mM Tris-HCl, pH 8.0, 10 mM EDTA 1% SDS) at 70°C for 10 min. DNA and chromatin de-crosslinking was done by incubation at 65°C for overnight in elution buffer. DNA elute was cleaned by PCR purification kit (Qiagen) and kept at −20°C until PCR or library prep for sequencing. To detect binding to *IL6* promoter regions, primer sets that detect each *PRDM1* consensus sequence was used for PCR. The PCR condition was as followed: 94°C for 5 min; 94°C for 30 s, 60°C for 30 s, and 72°C for 1 min for 40 cycles.

Set1: F- 5′-GCCTCAATGACGACCTAAGC-3′, R- 5′-ACGTCCTTTAGCATGGCAAG-3′,
Set2: F- 5′-GCGATGGAGTCAGAGGAAAC-3′, R- 5′-AGCTGAAGTCATGCACGAAG-3′,
Set3: F- 5′-CCTGGAGACGCCTTGAAGTA-3′, R- 5′-CTGTGAGCGGCTGTTGTAGA-3′,
Set4: F- 5′-TACAGGGAGAGGGAGCGATA-3′, R- 5′-GGCAGAAAGGGGGAGAATAC-3′,
Set5: F- 5′-AAATGCCCAACAGAGGTCAC-3′, R- 5′-AAACCAGACCCTTGCACAAC-3′,
Set6: F- 5′-CTCCCCCATTTTCATTTTCA-3′, R- 5′-TGGGGAAAGTGAGGTCATC-3′,
Set7: F-5′-TGAACATTTTATCATGAACACGAA-3′, R- 5′-CGTGCACTGTGATCCGTCTA-3′,
Set8: F- 5′-CGGTGAAGAATGGATGACCT-3′, R- 5′-GTGACCTCTGTTGGGCATTT-3′.

### Cloning IL-6 Promoter and Luciferase Reporter Assay

Primers to amplify the IL-6 area (forward, 5'-CGATATAGCCGAGCTGGAAG-3'; reverse, 5'- AAACCAGACCCTTGCACAAC-3') yield 932-bp amplicon. The PCR condition was as followed: 94°C for 5 min; 94°C for 30 s, 60°C for 30 s, and 72°C for 1 min 15 s for 30 cycles. *IL6* PCR product was cloned in pGL4.25 (Promega). 2 × 10^4^ HEK-293 cells were plated in 12-well culture plates in DMEM containing 10% FBS and 1% penicillin-streptomycin. *IL6* promoter luciferase reporter construct and *tk-Renilla* luciferase construct was transfected by Lipofectamine 2000 (Invitrogen). At 48 h post-transfection, transfected cells were lysed and assayed for both firefly and Renilla luciferase activity using the Dual-GLO Luciferase Assay System (Promega). Luciferase activity was measured using a luminometer (Perkin Elmer Victor3). The relative luciferase activity was calculated by normalization to the level of Renilla luciferase.

### T_FH_ Cell *in vitro* Differentiation

MO-DCs were differentiated and indicated siRNAs were transfected at day 5 during differentiation. Cells were further cultured for 2 days. MO-DCs were stimulated with lipopolysaccharides (LPS) (1.0 μg/ml) for 6 h and washed. Naïve CD4+ T cells were isolated from PBMCs by using an Easysep human naïve CD4+ T cell isolation kit (Stemcell technologies). 1.3 × 10^3^ of LPS pre-stimulated MO-DCs or unstimulated MO-DCs were co-cultured with 4 × 10^4^ naïve CD4+ T cells for 6 days. NC (negative control) was naïve CD4+ T cell alone and PC (positive control) was naïve CD4+ T cells with T_FH_ differentiation cocktails [CD2/3/28 activation beads (Miltenyl Biotec), IL-6 (50 ng/ml, R&D systems) and IL-12 (20 ng/ml, R&D systems)]. T_FH_ cells were analyzed by flow cytometry with a Fortessa (BD Biosciences). Fixable Viability Dye eFluor 506 (FVD, eBioscience) was used to exclude dead cells. For flow cytometry, antibodies were purchased from BioLegend (anti-CXCR5-APC: J252D4), eBioscience (anti-PD1-pacific blue: EH12.2H7 and anti-CD11c-Amcyan: B-ly6), and BD Bioscience (anti-IL-21-PE: 3A3-N2 and IFN-γ-APC/Cy7: B27).

### Quantitative RT-PCR (qRT-PCR)

Total RNA was extracted with Direct-zol RNA Micro Prep (Zymo Research,CA) and RNA samples were treated with DNase I to remove genomic Deoxyribonuclic acid (gDNA) contamination. cDNA was prepared with the iScript cDNA synthesis kit (Bio-Rad). Gene-specific primers were purchased from Taqman (Life Technologies) and qRT-PCR was performed with a Light cycler 480 II (Roche). Taqman primers: Hs00153368_m1 (*BCL6*), Hs00172187_m1 (*POLR2A*), Hs99999902_m1 (*RPLP0*), Hs00174131_m1 (*IL6*), Hs00153357_m1 (*PRDM1*), Hs00939763_g1 (*NonO*), Hs00175407_m1 (*CTSS*). Relative expression of a gene of interest to housekeeping gene was calculated by ΔCt or ΔΔCt.

### ELISA

To measure the cytokine secretion, supernatants from the MO-DCs were collected and the level of IL-6 was measured by human IL-6 ELISA kit (DuoSet ELISA kit, R&D Systems, Minnesota, USA). The lower level of detection for the assay was 4.68 pg/ml.

### Statistics

Statistical analysis was calculated and determined by a non-parametric Man-Whitney test in the Prism 6 (Graphpad software). *P* < 0.05 were considered significant.

## Results

### NonO Is a PRDM1 Binding Protein in MO-DCs

PRDM1 is known to regulate gene expression by recruitment of chromatin modifiers, including histone deacetylases (HDACs), lysine-specific demethylase1 (LSD1), protein arginine methyltransferase (PRMT5), and euchromatic histone-lysine N-methyltransferase 2 (EHMT2, also known as G9a) in PCs and primordial germ cells (16–19). PRDM1 also recruits polycomb repressive complex 2 (PRC2) by directly binding the enhancer of zeste homolog 2 (*Ezh2*) domain of PRC2 in murine plasmablasts (26). We investigated whether PRDM1 recruits the same chromatin modifiers in MO-DCs. Binding of HDAC1, HDAC 2, PRMT5 or G9A to PRDM1 was assessed by Co-IP; however, no significant binding of any of those molecules to PRDM1 in MO-DCs was found (Figure S1B and data not shown).

To identify binding proteins of PRDM1 in a non-biased way, relative and absolute quantitation (iTRAQ) MS was performed on the nuclear fraction of MO-DCs immunoprecipitated by PRDM1 antibody. Compared to the fraction immunoprecipitated by control IgG, 39 proteins were pulled-down specifically by the anti-PRDM1 antibody (cutoff >1.5-fold) (Table S1). Consistent with the Co-IP data, there were no HDACs or other known chromatin modifiers identified by mass spectrometry. Thus, PRDM1 does not recruit detectable chromatin modifiers for regulation of target gene expression in MO-DCs.

**Figure 1:**
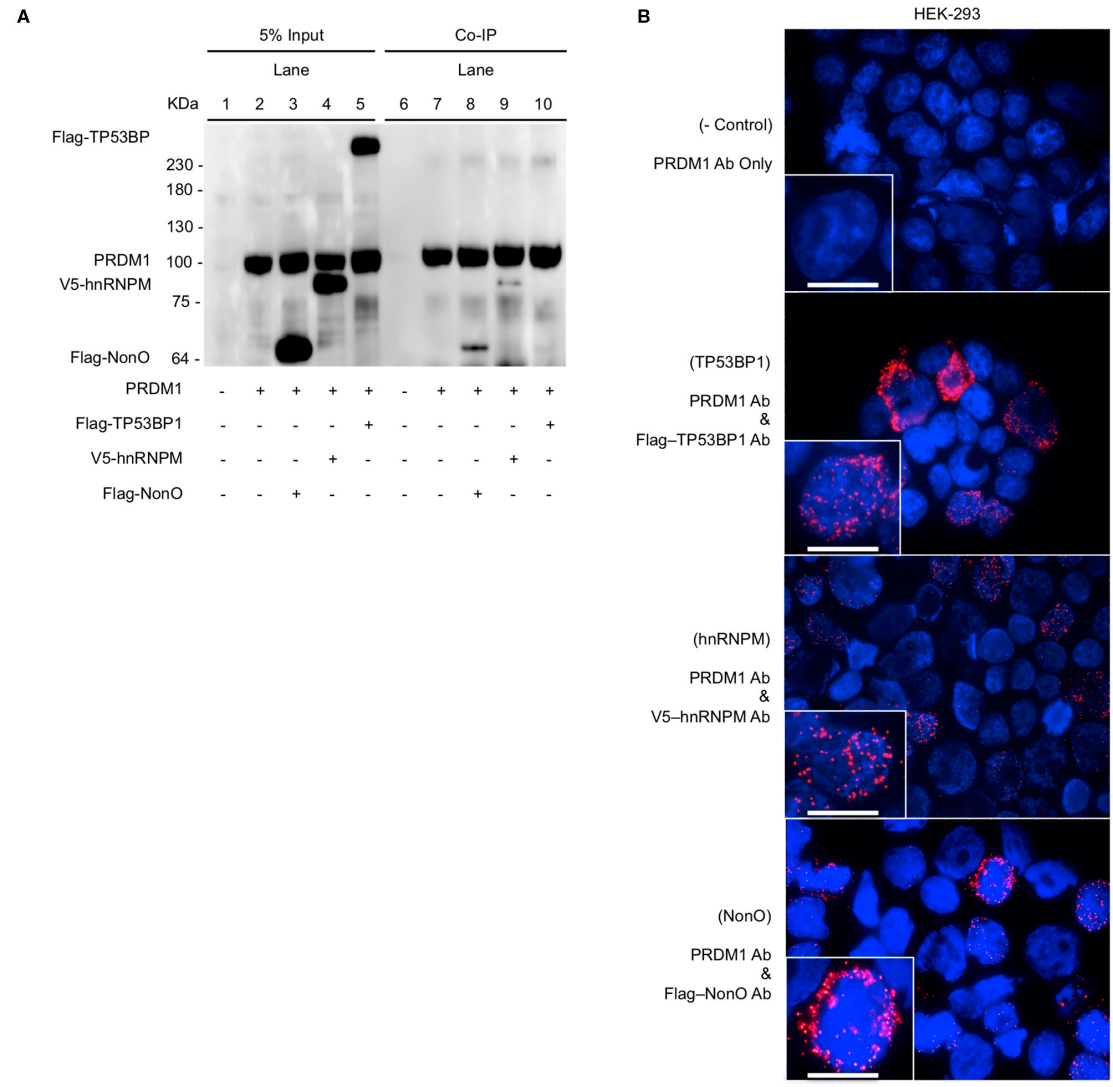
Binding of PRDM1 and candidate proteins in HEK-293 cells. HEK-293 cells were transfected *PRDM1* alone or together with *Flag-NonO, Flag-TP53BP1*, or *V5-hnRNPM* expression vector. Binding between PRDM1 and each candidate proteins was detected by Co-IP and PLA. **(A)** Nuclear fraction was immunoprecipitated with anti- PRDM1 antibodies and immunoblotting was performed with anti-PRDM1, Flag-NonO, Flag-TP53BP1, or V5-hnRNPM antibody. A representative image from two independent experiments is shown. **(B)** Binding between PRDM1 and candidate proteins was visualized by PLA (red color) and nuclei were stained with DAPI (blue). Top panel; technical negative control PLA (PRDM1 antibody alone), other panels; detection of PLA (PRDM1 with Flag or V5 Ab). Scale bar = 10 μm. A representative image from three independent experiments. Co-IP, co-immunoprecipitation (Co-IP); PLA, proximity ligation assay.

Among the PRDM1-associated proteins identified by MS, we chose three molecules, NonO, Tumor Protein P53 Binding Protein 1(TP53BP1), and Heterogeneous Nuclear Ribonucleoprotein M (hnRNPM), as candidate co-regulators of PRDM1 due to their known transcriptional regulatory functions. To verify the interaction between those three proteins and PRDM1, Co-IP was performed in HEK-293 cells. Since HEK-293 cells do not express PRDM1 endogenously, they were transiently transfected with vectors encoding *PRDM1* and *Flag-NonO, Flag-TP53BP1*, or *V5-hnRNPM*. As expected, PRDM1 were not detected in input of non-transfected HEK-293 (Figure 1A, lane 1) and immunoprecipitated proteins from HEK-293 cell nuclear extract (PRDM1-negative) did not display any of the three proteins (NonO, TP53BP1, and hnRNPM) by Western blot (Figure 1A, lane 6). In contrast, anti-FLAG and anti-V5 immunoblotting, which detect Flag-NonO, Flag-TP53BP1, and V5-hnRNPM, showed an association between PRDM1 and NonO and hnRNPM in PRDM1-transfected cells (Figure 1A, lane 8 and 9) but no interaction between PRDM1 and TP53BP1 (Figure 1A, lane10).

PLA was used to verify these interactions in HEK-293 cells. PLA is an antibody-based detection technique that permits the assessment of colocalization between two proteins within < ~40 nm distance in a cell (27, 28). PLA complexes are depicted as red puncta and each punctum represents an interaction between PRDM1 and a candidate molecule. PLA-positive red clusters were not detected in the technical control, which included incubation with only anti-PRDM1 antibody (Figure 1B, top panel). All three candidates, NonO, TP53BP1, and hnRNPM led to PLA positive clusters with PRDM1 (Figure 1B). The signals from hnRNPM and NonO were predominantly nuclear while signal from TP53BP1 was detected in the cytoplasm, suggesting that the interaction of hnRNPM and NonO with PRDM1 may be involved in regulation of gene expression while an interaction of TP53BP1 and PRDM1 may regulate pathways in the cytoplasmic compartment. This observation explains the lack of association of TP53BP1 and PRDM1 in the Co-IP of nuclear extracts.

**Figure 2:**
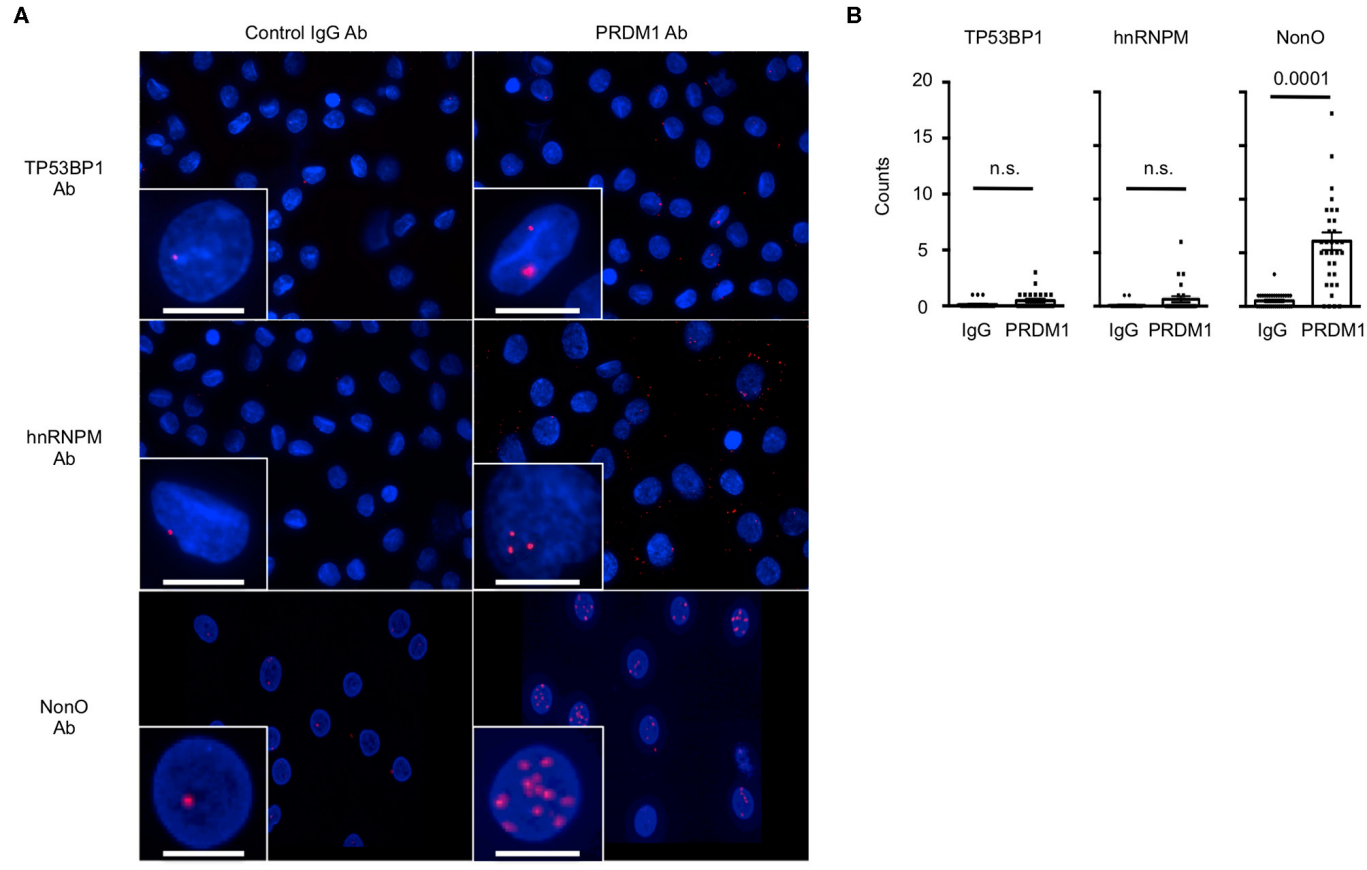
The interaction of NonO and PRDM1 in the nucleus of MO-DCs. Binding of candidate proteins with PRDM1 in primary MO-DCs was measured by PLA. **(A)** MO-DCs were incubated with normal rabbit IgG (left column) or with anti-PRDM1 antibodies (right column) together with anti-TP53BP1, anti-hnRNPM, or anti-NonO antibodies. Their proximal interaction was assessed by PLA and visualized as red dots. Nucleus was stained with DAPI (blue). Each Z-Stacks maximum intensity projection image is a representative from three independent experiments and captured 60x magnification. Scale bar = 10 μm. **(B)** Quantification of the PLA signal on MO-DCs is represented. PLA signals in each nucleus were quantified by Imaris software. In the plot, horizontal bars indicate the mean with SEM and each dot represents counts of individual nucleus (*n* = 40–50). Significance determined by unpaired *t*-test.

We further validated these results in MO-DCs, in which we did not need to overexpress PRDM1. No PLA signals were detected in MO-DCs with any single primary antibodies and normal IgG (Figure 2A, left panel). As expected, PLA signals were detected between PRDM1 and NonO in the nucleus of MO-DCs (Figure 2A, the bottom of right panel). There was no significant signal detected with hnRNPM and TP53BP1 in either the nucleus or the cytoplasm (Figure 2A, the top and middle of right panel). Quantitative analysis of the PLA signal between PRDM1-candidate proteins and negative control-IgG confirmed specific PLA signals between PRDM1 and NonO (Figure 2B, right graph).

Additionally, PRDM1 in nuclear extracts coprecipitated with NonO but not with hnRNPM and TP53BP1 (Figure S2). These inconsistent results obtained from primary MO-DCs and HEK-293 cells are likely due to the overexpression of PRDM1 in HEK293 cells. The data obtained from MS, Co-IP and PLA confirmed that NonO is a novel PRDM1 binding protein in the nucleus of MO-DCs.

### NonO Co-regulates Expression of IL-6 in MO-DCs

**Figure 3:**
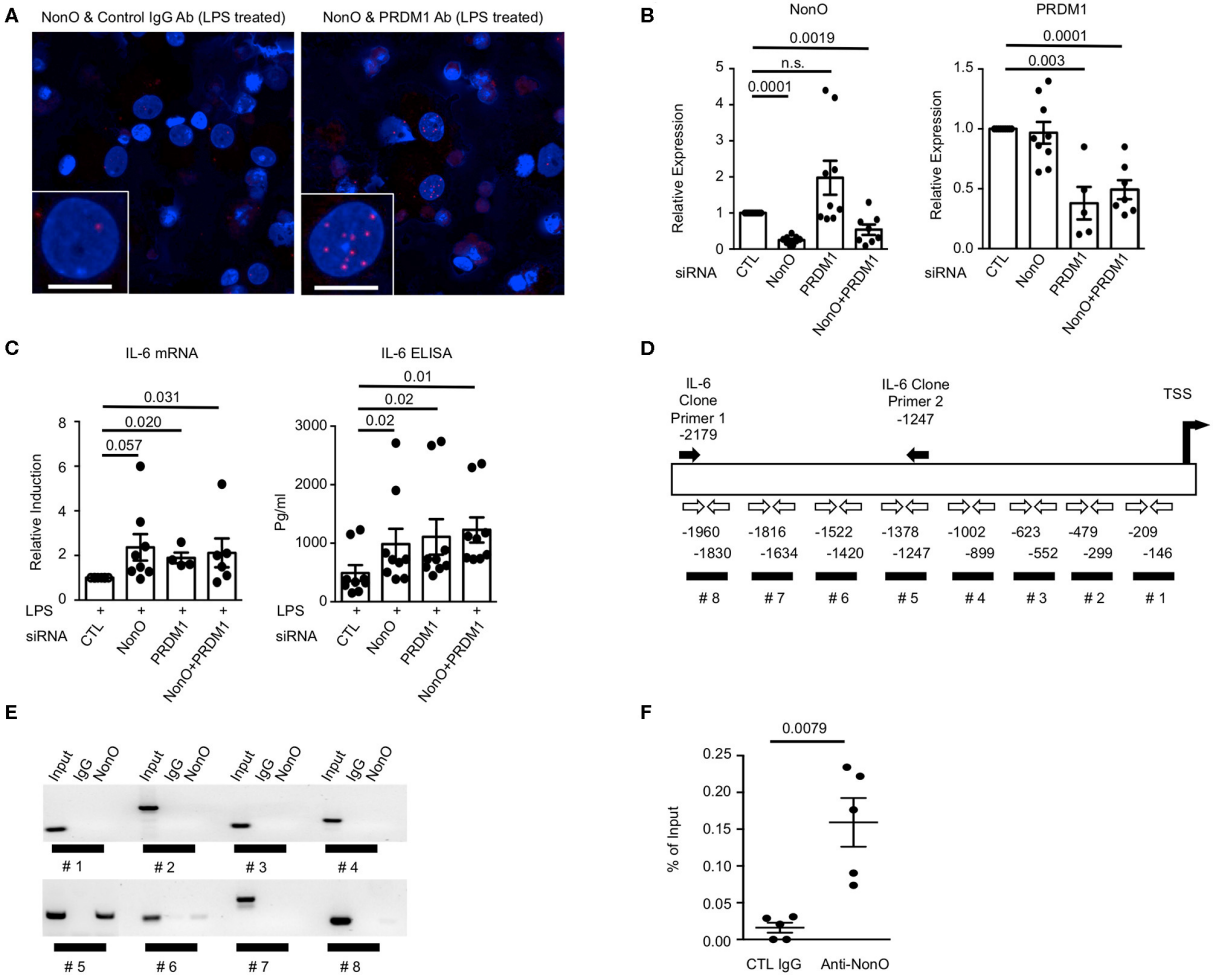
NonO-dependent regulation of IL-6 by PRDM1 in MO-DCs. **(A)** MO-DCs were stimulated with LPS for 6 h and stained with normal rabbit IgG + anti-NonO antibodies (left column) or with anti-PRDM1 antibodies + anti-NonO antibodies (right column). Their proximal interaction was assessed by PLA and visualized as red dots. Nucleus was stained with DAPI (blue). Each Z-Stacks maximum intensity projection image is a representative from three independent experiments and captured 60x magnification. Scale bar = 10 μm. **(B)** To knock down NonO, PRDM1, and both (NonO and PRDM1) expression, indicated siRNA or control siRNA was transfected into MO-DCs and NonO and PRDM1 expression level were quantified by qRT-PCR. Bar graph is a mean ± SEM (*n* = 9). Significance determined by Man-Whitney test. **(C)** Indicated siRNA or control siRNA transfected MO-DCs were cultured with or without LPS (1 μg/ml) for 6 h, and total RNA was purified. Level of *IL6* was measured by qRT-PCR and relative induction was calculated by normalization to the level of LPS stimulated control. Supernatant concentrations of IL-6 obtained from the cultures were measured using enzyme-linked immunosorbent assay (ELISA). Bar graph is a mean ± SEM (*n* = 9). Significance determined by Mann Whitney test. **(D)** Diagram of human *IL6* promoter region with indication of putative PRDM1 binding site (black bar #1–#8) and PCR primers (open arrow). Primer set for *IL6* promoter cloning is designated as black arrows. ChIP was performed with anti-NonO antibodies or control IgG from MO-DCs. **(E)** PCR result was visualized in agarose gel. Binding of NonO to PRDM1 consensus sequences within the *IL6* promoter were assessed by each primer set (indicated in **C**). **(F)** To quantify the binding of NonO to #5 region, qPCR was performed and calculated by the percent of input. The graph is a mean ± SEM (*n* = 5). Significance determined by Mann Whitney test.

Knowing that PRDM1 and NonO interact in the nucleus of MO-DCs, we further investigated whether NonO participates in the transcriptional function of PRDM1. Previous data showed that the level of the proinflammatory cytokine IL-6 was negatively regulated by PRDM1 in DCs in response to LPS stimulation (11, 14, 29). If NonO is required for PRDM1-mediated suppression of target gene expression, NonO-deficiency could lead to an increase in the level of IL-6 after LPS stimulation. We confirmed the binding of PRDM1 and NonO in MO-DCs after LPS stimulation (Figure 3A). Next, *NonO* or *PRDM1* targeting siRNA or scrambled control siRNA was transfected into MO-DCs and the level of *IL-6* was measured. Effective knockdown of NonO, PRDM1 or both was achieved; about 50% of either *NonO* or *PRDM1* mRNA was present in *NonO, PRDM1* or both *NonO and PRDM1*-siRNA compared to control siRNA transfected MO-DCs (Figure 3B). *NonO* expression was unchanged in PRDM1-deficient MO-DCs and PRDM1 expression was unchanged in NonO-deficient MO-DCs. To investigate whether *IL-6* expression is regulated by the level of NonO, PRDM1 or both, the level of *IL-6* was measured in the basal state and at 6 h after LPS stimulation. The basal level of *IL-6* mRNA and IL-6 protein in the supernatant were minimal and no change was detected with knock down of NonO, PRDM1, or both (Figure S3). In contrast, following LPS stimulation, IL-*6* induction (both transcripts and protein in the supernatant) was increased in siRNA-transfected MO-DCs with NonO, PRDM1, or both compared to control siRNA-transfected MO-DCs (Figure 3C). There was no synergistic effect observed in double-knock down MO-DCs, suggesting PRDM1 and NonO are in a same regulatory pathway. These data demonstrate that NonO-deficiency and PRDM1-deficiency lead to the up-regulation of IL-6 in LPS stimulated MO-DCs.

Previous reports demonstrated that NonO can regulate gene expression by binding to promoter regions (transcriptional regulation) or by binding to mRNA (post-transcriptional regulation) (30–32). Therefore, we investigated the mechanism of NonO-mediated IL-6 expression in MO-DCs. First, binding of NonO to PRDM1-binding sites in the *IL6* promoter area was investigated. A search for the PRDM1 binding motif in the *IL6* promoter area revealed eight potential PRDM1 binding sites which contain the consensus sequence (A/C)AG(T/C)GAAAG(T/C)(G/T) or (A/C)AG(T/C)GAAAT(T/C)(G/T) within 2,000 bp upstream from transcription start site (TSS) (33) (Figure 3D, #1–#8). We first performed ChIP-PCR using anti-NonO antibody or control antibody and the binding of NonO to PRDM1 binding sites in the *IL6* promoter region was assessed. The ChIP efficiency was optimized by detection of *P4H*α*1*, a known target gene of NonO in DNA precipitated with anti-NonO antibody compared to control IgG (data not shown) (32). We performed PCR analysis with primer sets at multiple sites throughout the IL-6 gene; region #5 (−1,247~–1,378 bp TSS) was significantly enriched in DNA precipitated with anti-NonO antibody (Figure 3E). Thus, the #5 region is recognized by NonO. The percent of input (%IP) was calculated from the quantitative PCR (qPCR) and 2–5 fold more enrichment was observed with anti-NonO antibody compared to control IgG (Figure 3F). To confirm whether PRDM1 can bind to the same recognition sequence as NONO, we performed ChIP with anti PRDM1 antibody. We could detect enrichment of PRDM1-binding to IL-6 promoter at region (#5), but the difference between control IgG and PRDM1 was not significant (Figure S4). Taken together, NonO-PRDM1 complexes are recruited to *IL6* promoter region to suppress IL-6 expression in MO-DCs.

**Figure 4:**
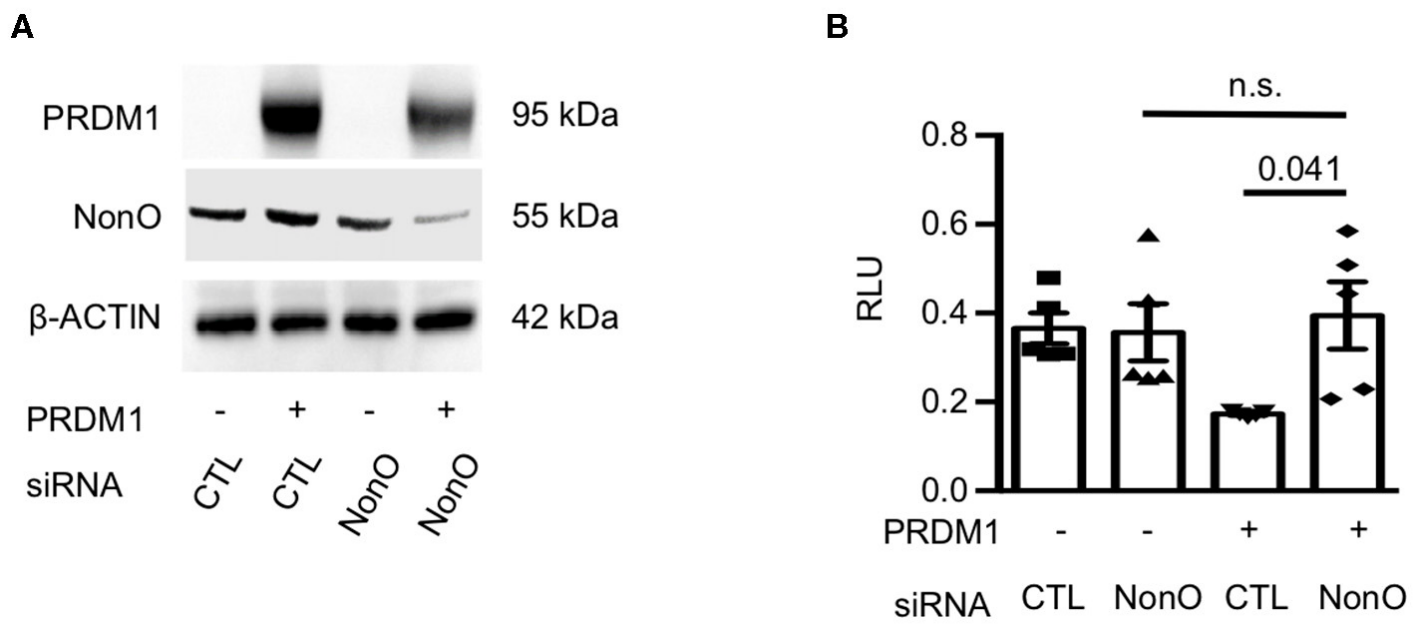
NonO regulates IL-6 promoter activity. **(A)** HEK-293 cells were transfected with NonO siRNA or *PRDM1* expressing plasmid. The knockdown efficiency of siRNA and PRDM1 level was verified by immunoblott analysis. β- actin was used as a loading control. Gel image is a representative from two independent experiments. **(B)** The *IL6* promoter activity under indicated conditions was determined by luciferase reporter gene analysis. pGL4.25 were used as control vectors. Mean values of relative luciferase unit (RLU; normalized on Renilla luciferase) from three independent experiments. Bar is a mean ± SEM (*n* = 4). Significance determined by unpaired *t*-test.

To further investigate whether NonO can regulate the transcription of *IL6*, a luciferase reporter assay was performed. Since ChIP-PCR results showed that NonO/PRDM1 binding was enriched in the proximal region [−1.3~–2.2 kb] of the *IL6* gene promoter, we engineered an *IL6* promoter-Luc plasmid (pGL4.25) containing NonO/PRDM1-binding region of human genomic DNA. To modulate the level of PRDM1 and NonO in the HEK-293 cell line, HEK-293 cells were transfected with a PRDM1 expressing plasmid with control siRNA or with *NonO* siRNA. *NonO* siRNA led to a 30–60% decrease in NonO protein levels compared to levels in control siRNA transfected HEK-293 cells (Figure 4A). As expected, PRDM1 suppressed *IL6* promoter activity; this suppressive effect was abrogated by a decrease in NonO (Figure 4B). These results demonstrate that NonO functions to enable transcriptional repressor of the *IL6* gene by PRDM1. There is no significant change in *IL6* promoter activity by NonO deficiency without PRDM1 expression; thus, the regulatory mechanism of NonO depends on the PRDM1 expression level.

### NonO-PRDM1 Complexes Regulate the Generation of T_FH_-Like Cells

**Figure 5:**
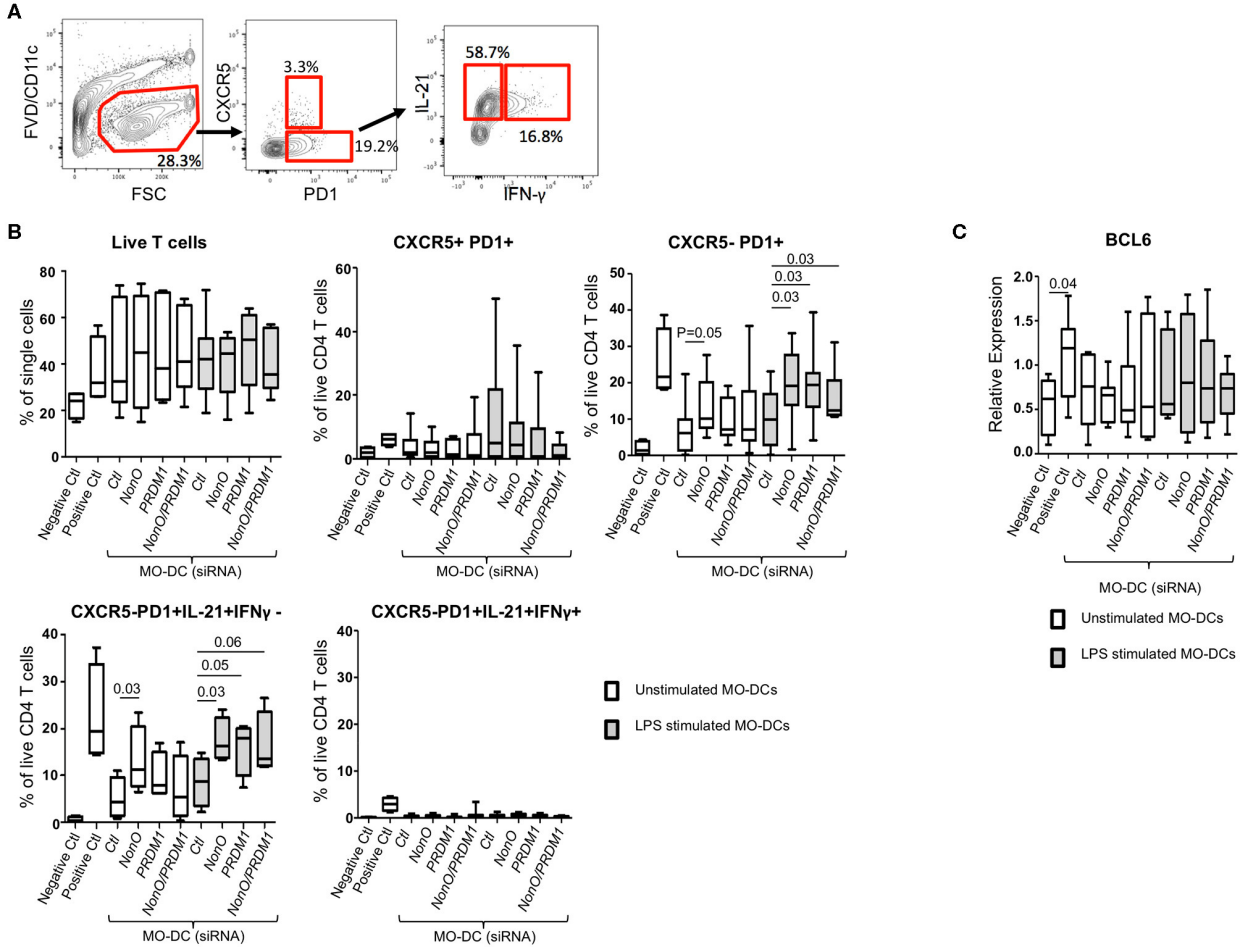
PRDM1- or NonO-deficient MO-DCs induce increased T_FH_ differentiation. Allogenic culture of naïve CD4+ T cells and MO-DCs was set up to induce T_FH_ differentiation. Expression levels of PRDM1 or NONO in MO-DCs were modulated by transfection with siRNAs before co-culture. After 6-days culture, T_FH_ cell differentiation was measured by IL-21, IFNγ, CXCR5, and PD1 expression by flow cytometry. **(A)** A representative flow image. **(B)** Dead cells were excluded using Fixable Viability Dye eFluor 506, and the percentage of live CD4^+^ T cells was calculated. T_FH_ -like cells (CXCR5+/PD1+), CXCR5- helper T cells (CXCR5-/PD1+), and cytokine expressing helper T cells (CXCR5-/PD1+/ IL21+/ IFN-γ-, and CXCR5-/PD1+/IL21+/ IFN-γ+) were calculated and plotted. Co-culture of T cells with LPS pre-stimulated MO-DCs was indicated with gray filled box and culture with unstipulated MO-DCs was indicated with open box. Negative control is naïve CD4+ T cell alone and Positive control is naïve CD4+ T cells with CD2/3/28 activation beads IL-6 (50 ng/ml) and IL-12 (20 ng/ml). In the Box-and-Whisker plot, horizontal bars indicate the median, boxes indicate 25–75th percentile, and the whiskers indicate 10 and 90th percentile. Four independent experiments (*n* = 9). Significance determined by Mann Whitney test. **(C)** *BCL6* expression was quantified by qRT-PCR. Relative expression was calculated to the level of housekeeping gene, *POLR2A*. In the Box-and-Whisker plot, horizontal bars indicate the median, boxes indicate 25–75th percentile, and the whiskers indicate 10 and 90th percentile. Three independent experiments (*n* = 9). Significance determined by Mann Whitney test.

IL-6 production is one of key factors for murine follicular helper T cell (T_FH_) differentiation, and an increased production of IL-6 in DCs leads to an expansion of T_FH_ in *Prdm1* CKO mice (11, 14, 29). To address the function of PRDM1-deficiency and NonO-deficiency in DCs on the differentiation of CD4^+^ T_FH_ cells, unstimulated or LPS pre-stimulated MO-DCs were co-cultured with naïve CD4+ T cells. After co-culture, surface phenotype and cytokine production by T cells were investigated by flow cytometry (Figure 5A). After co-culture, live CD4+ T cells were identified by exclusion of FVD-positive (dead cells) and CD11c-positive (MO-DCs). There was no difference in the expansion of T cells, and CXCR5-positive T cells were not strongly induced in any culture condition (data not shown). Interestingly, LPS-stimulated PRDM1-deficient or NonO-deficient MO-DCs induced a higher percent of IL-21+CXCR5-PD1+ cells compared to control siRNA-treated MO-DCs (Figure 5B). NonO-deficient MO-DCs also induced higher percent of IL-21+CXCR5-PD1+ T cells even in the absence of LPS stimulation, but this effect was not observed in PRDM1- or double deficient MO-DCs (Figure 5B). We do not know the mechanism, but NonO may regulate other regulatory molecules which positively regulate T cell differentiation or survival. We also compared IFN-γ production in T cells, but none of T cells were IFN-γ-positive (Figure 5B). To confirm the IL-21-producing T cells are T_FH_ cells, we measured the induction of B-Cell Lymphoma 6 (BCL6) which is a master transcription factor of Tfh cells (34). The *BCL6* induction was small and not significantly higher than negative control group, and there was no difference of *BCL6* levels among the groups (Figure 5C). Therefore, NonO, PRDM1, or PRDM1/ NonO deficient MO-DCs generate more IL-21 producing T_FH_-like cells_._ No synergistic effects of PRDM1 and NonO were observed in IL-21 producing T_FH_-like cell differentiation. Increased production of IL-6 might contribute to this alteration.

### NonO-PRDM1 Interaction Does Not Regulate IL-6 Expression in Myeloma Cells

**Figure 6:**
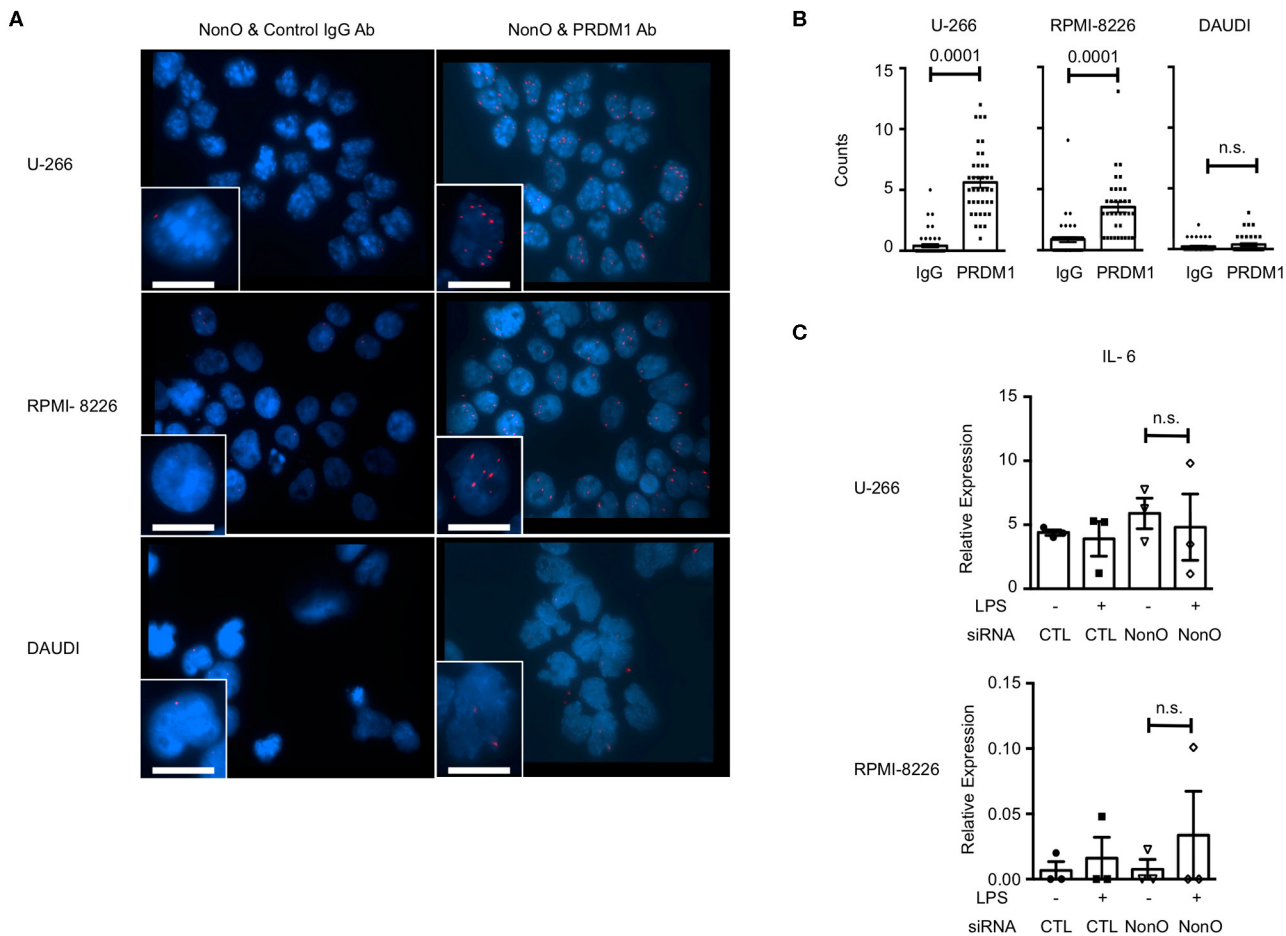
NonO binds with PRDM1 but does not regulate IL-6 in myeloma cells. **(A)** Binding between PRDM1 and NonO in human myeloma cells was visualized by PLA (red color) and nuclei were stained with DAPI (blue). Left columns are control groups of PLA (anti-NonO and control IgG) and right columns are experimental groups (anti-NonO and anti-PRDM1). Scale bar = 15 μm. Images were taken at 60x magnification. A representative image from three independent experiments. **(B)** PLA signals in each nucleus were quantified by Imaris software (*n* = 30–40). Significance determined by Mann Whitney test. **(C)** 48 h *NonO* or control siRNA post transfection, cells were cultured with or without LPS (1 μg/ml) for last 6 h and level of *IL-6* measured by qRT-PCR. Bar graph is a mean ± SEM (*n* = 3). Significance determined by unpaired *t*-test.

Since plasma cells express a high level of PRDM1 and secrete IL-6, we wanted to know whether PRDM1 and NonO regulate IL-6 in the human myeloma B cell lines, U-266, and RPMI-8226 since both cell lines express a high level of PRDM1 and NonO (Figure S5A). Daudi, a non-myeloma B cell line with a high level of NonO but not PRDM1, was used as a negative control. Using the PLA assay, we found a NonO and PRDM1 interaction in U-266 and RPMI-8226 in nucleus but not in Daudi (Figures 6A,B). Next, we tested whether NonO participates in PRDM1-mediated IL-6 production in these cells. NonO knockdown mediated by siRNA was sufficient to decrease the NonO expression level in both U-266 and RPMI-8226 cells (Figure S5B). In contrast to MO-DCs, there is high level of endogenous IL-6 expression in myeloma cells which was not further increased by stimulation with LPS, and no significant induction of IL-6 when NonO levels were decreased (Figure 6C). Similarly, PRDM1-deficiency did not increase the expression level of basal or LPS stimulated *IL-6* mRNAs in U-266 (Figure S5B). Hence, in myeloma cells, NonO could be recruited to a PRDM1 complex but no PRDM1-mediated regulatory effects on IL-6 expression by NonO-deficiency and PRDM1-deficiency were observed.

## Discussion

PRDM1 is a transcription factor that is expressed in multiple immune cells including myeloid cells (22, 35, 36). A proper expression is required for terminal differentiation of myeloid lineage cells (36). It is also involved in immune homeostasis and an insufficient level of PRDM1 in DCs leads to a breakdown in immune tolerance in mice (11). However, little is known about the molecular mechanisms behind its function in myeloid cells. Previous reports suggest that the suppressive function of PRDM1 depends on its cofactors. In order to identify cofactors of PRDM1 in MO-DCs, we performed both Co-IP and MS experiments. Binding of putative cofactors to PRDM1 was assessed by PLA. These results show that a PRDM1-NonO interaction occurs which is localized to the nucleus. The interaction can be observed even after LPS stimulation. Together, these molecules regulate IL-6 expression. The regulatory function on IL-6 is observed only in MO-DCs but not in human myeloma cells. It is well-accepted that IL-6 positively regulates IL-21 production in T cells, and the activation of STAT3 which is downstream of IL-6R signaling pathway is required for transcriptional activation of IL-21 (37–39). Deficiency of either NonO or PRDM1 in MO-DCs also leads to an expansion of IL-21-producing T_FH_-like cells *in vitro*, suggesting that a proper expression of both NonO and PRDM1 is required for the proinflammatory function of MO-DCs. Interestingly, NonO might regulate function of MO-DCs which induces helper T cell differentiation without LPS stimulation. This alteration is not by IL-6 expression. Indeed, NonO itself can regulate gene expression in multiple mechanisms, including chromatin remodeling, transcriptional regulation, or post-transcriptional regulation (30, 40). We do not know what molecules are targeted by NonO in MO-DCs under homeostatic condition, and this needs to be investigated.

It has been previously shown by us and others that allele specific effects on gene expression may be cell lineage specific. This has been noted to relate to polymorphism-generated acquisition or loss of transcription factor binding sites. Indeed, a PRDM1 SNP that is associated with increased risk for developing SLE is operative in myeloid cells. The risk allele has a KLF4 binding site, which leads to decreased gene expression. As B cells do not express KLF4, there is no allele-specific change in expression of PRDM1 in B cells (23). Here we show differential effects of transcription factors in myeloid cell and B cells, even when both lineages express the transcription factors and the target gene. The mechanism for this requires elucidation. This could result from different chromatin accessibility of target genes, from multi-protein complexes including other unidentified transcription factors or from inhibitors of transcription factor binding to some regulatory regions of the target gene. In our case, the difference is not due to the accessibility of a PRDM1-NonO complex to the genomic area since both cells readily express IL-6. The functional difference may depend on additional cell type-specific co-regulators in myeloid cells and B cells. Indeed, NonO is known to be present in multi-protein complexes in the nucleus. In an *in vitro* system, PU.1 (Spi-1) binds to NonO and impedes NonO binding to RNA (41); the same may relate to DNA binding although it was not studied in that report. PU.1 is highly expressed in myeloid cells and in early stage of B cells, but its expression is suppressed in plasma cells (42) and completely negative in myeloma cells (43). Therefore, PU.1 might be a candidate regulator in regulation of function of PRDM1/NonO complex.

Additional remaining questions are what genes other than IL-6 are regulated by PRDM1 in conjunction with NonO and what genes are regulated by PRDM1 independent of NonO. CTSS is another gene which is negatively regulated by PRDM1 in MO-DCs (14), but CTSS expression was not changed by knock down of NonO (Figure S6). This suggests PRDM1 regulates gene expression in both a NonO-dependent and NonO-independent manner in the same cell, with CTSS as one example of NonO-independent regulation of PRDM1. All of these questions are critical for understanding the mechanism of gene regulation by PRDM1 and function in MO-DCs.

In summary, our data demonstrated that NonO is a co-factor of PRDM1 and recruitment of NonO by PRDM1 is required for transcriptional regulatory function of PRDM1 in MO-DCs. The absence of PRDM1 or NonO increased the expression of IL-6 which is a positive regulator of IL-21-producing T_FH_-like cell differentiation. NonO interaction to PRDM1 regulates gene transcription in MO-DCs but not in myeloma cells, representing a new paradigm for exploring lineage specific effects of transcriptional regulators.

## Data Availability Statement

All datasets generated for this study are included in the article/Supplementary Material.

## Author Contributions

SK designed and conceived the study and analyzed and interpreted the data. SJ performed the experiment of Co-IP and mass spec and helped analyze the data. KL performed most experiments, data analysis, and helped interpret the data. HT performed gene expression studies and cell culture. KL and SK wrote the manuscript. All contributing authors have agreed to the submission of this manuscript for publication.

## Conflict of Interest

The authors declare that the research was conducted in the absence of any commercial or financial relationships that could be construed as a potential conflict of interest.

## Supplementary Material

Figure S1 **(A)** A representative flow image of MO-DCs. Day 7 of MO-DCs (non-adherent cells) and adherent cells were collected and viability and the purity were investigated by exclusion of live/dead fixable marker, FVD and HLA-DR and CD209 antibodies. Purity of MO-DC was calculated by percentage of live HLA-DR/CD209 double positivity. **(B)** Assessment of HDAC binding to PRDM1 by Co-IP. Nuclear fraction of MO-DCs was immunoprecipitated with anti- PRDM1 antibodies or control IgG and immunoblotting was performed with anti-HDAC1 or anti-HDAC2 antibodies. A representative image from two independent experiments is shown.

Figure S2 Binding of PRDM1 with NonO, hnRNPM, and TP53BP1 by Co-IP. Nuclear fraction of MO-DCs were incubated with ant-PRDM1 or control IgG and immunoblotting was performed with anti-NonO, hnRNPM, or TP53BP1 antibodies. Input is an unfractionated total nuclear extract. A representative image of two independent experiments.

Figure S3 The basal level of *IL-6* transcript and protein were measured by qRT-PCR and ELISA. NonO, PRDM1, and both (NonO and PRDM1) siRNA or control siRNA transfected MO-DCs were cultured without LPS (1 μg/ml) for 6 h, and total RNA was purified. Relative level of *IL-6* was measured by qRT-PCR and normalized to the level of housekeeping gene, *POLR2A*. Supernatant concentrations of IL-6 obtained from the cultures were measured using ELISA. Bar graph is a mean ± SEM (*n* = 9). Significance determined by Mann Whitney test.

Figure S4 Assessment of PRDM1 binding to *IL6* promoter regions by ChIP-qPCR. To test PRDM1 binding to *IL6* promoter, ChIP was performed. Nuclear fraction of MO-DCs and ChIP was performed by anti-RPDM1 or control IgG as described in material and method. PCR **(A)** or qPCR **(B)** was performed to assess binding of PRDM1 by primers described in material methods. #1–#8 indicates each region including putative PRDM1 binding sites in IL6 promoter. **(A)** is a representative image of three independent experiments. **(B)** To quantify the binding of PRDM1 to #5 region, qPCR was performed and calculated by the percent of input. Each dot represents an individual sample and the bar represents the mean ±SEM (*n* = 3). Significance determined by Mann Whitney test.

Figure S5 Expression of *IL6* by NonO or PRDM1 in myeloma cells. **(A)** NonO expression was knock down by transfection of anti-NonO siRNA or scrambled control siRNA. After transfection, relative level of *NonO* was measured by qRT-PCR and normalized to the level of housekeeping gene, *POLR2A*. **(B)** To knock down the PRDM1 expression, anti-*PRDM1* siRNA, or control siRNA was transfected to U266 cells and *PRDM1* level was measured by qRT-PCR. U266 cells transfected with control or anti-PRDM1 siRNA was cultured with or without LPS (40 μg/ml) for 6 h. Relative level of *PRDM1, IL6* was normalized to the level of *POLR2A*. Each dot represents an individual sample and the bar is the mean ± SEM. Significance determined by Mann Whitney test.

Figure S6 The level of *CTSS* was measured by qRT-PCR. NonO, PRDM1, and both (NonO and PRDM1) siRNA or control siRNA transfected MO-DCs were cultured with or without LPS (1 μg/ml) for 6 h, and total RNA was purified. Relative level of *CTSS* was measured by qRT-PCR and normalized to the level of housekeeping gene, *RPLP0*. Then, relative induction was calculated by normalization to the level of control. Bar graph is a mean ± SEM (*n* = 6). Significance determined by Mann Whitney test.

Table S1 Mass spectrometric identification of candidate PRDM1 binding proteins in MO-DCs. The comparative analysis of peptide and protein quantification in normal IgG and PRDM1 of PRDM1-sufficient MO-DCs are subjected through iTRAQ-based quantitative proteomics with cutoff >1.5-fold. The experiment was repeated two times. iTRAQ, isobaric tags for relative and absolute quantitation; MO-DCs, monocyte derived-dendritic cells.

## References

[1] Keller AD Maniatis T. Identification and characterization of a novel repressor of beta-interferon gene expression. Genes Dev. (1991) 5:868–79. 10.1101/gad.5.5.868 1851123

[2] Keller AD Maniatis T. Only two of the five zinc fingers of the eukaryotic transcriptional repressor PRDI-BF1 are required for sequence-specific DNA binding. Mol Cell Biol. (1992) 12:1940–9. 10.1128/MCB.12.5.1940 1569931 PMC364364

[3] Fairfax KA Corcoran LM Pridans C Huntington ND Kallies A Nutt SL. Different kinetics of blimp-1 induction in B cell subsets revealed by reporter gene. J Immunol. (2007) 178:4104–11. 10.4049/jimmunol.178.7.4104 17371965

[4] Chang DH Cattoretti G Calame KL. The dynamic expression pattern of B lymphocyte induced maturation protein-1 (Blimp-1) during mouse embryonic development. Mech Dev. (2002) 117:305–9. 10.1016/S0925-4773(02)00189-2 12204275

[5] Robertson EJ Charatsi I Joyner CJ Koonce CH Morgan M Islam A. Blimp1 regulates development of the posterior forelimb, caudal pharyngeal arches, heart and sensory vibrissae in mice. Development. (2007) 134:4335–45. 10.1242/dev.012047 18039967 PMC7116377

[6] Angelin-Duclos C Cattoretti G Lin KI Calame K. Commitment of B lymphocytes to a plasma cell fate is associated with Blimp-1 expression *in vivo*. J Immunol. (2000) 165:5462–71. 10.4049/jimmunol.165.10.5462 11067898

[7] Shaffer AL Lin KI Kuo TC Yu X Hurt EM Rosenwald A. Blimp-1 orchestrates plasma cell differentiation by extinguishing the mature B cell gene expression program. Immunity. (2002) 17:51–62. 10.1016/S1074-7613(02)00335-7 12150891

[8] Shapiro-Shelef M Lin KI McHeyzer-Williams LJ Liao J McHeyzer-Williams MG Calame K. Blimp-1 is required for the formation of immunoglobulin secreting plasma cells and pre-plasma memory B cells. Immunity. (2003) 19:607–20. 10.1016/S1074-7613(03)00267-X 14563324

[9] Turner CA Mack DH Davis MM. Blimp-1, a novel zinc finger-containing protein that can drive the maturation of B-lymphocytes into immunoglobulin-secreting cells. Cell. (1994) 77:297–306. 10.1016/0092-8674(94)90321-2 8168136

[10] Zhou XJ Lu XL Lv JC Yang HZ Qin LX Zhao MH. Genetic association of PRDM1-ATG5 intergenic region and autophagy with systemic lupus erythematosus in a Chinese population. Ann Rheum Dis. (2011) 70:1330–7. 10.1136/ard.2010.140111 21622776

[11] Kim SJ Zou YR Goldstein J Reizis B Diamond B. Tolerogenic function of Blimp-1 in dendritic cells. J Exp Med. (2011) 208:2193–9. 10.1084/jem.20110658 21948081 PMC3201204

[12] Smith MA Wright G Wu J Tailor P Ozato K Chen X. Positive regulatory domain I (PRDM1) and IRF8/PU.1 counter-regulate MHC class II transactivator (CIITA) expression during dendritic cell maturation. J Biol Chem. (2011) 286:7893–904. 10.1074/jbc.M110.165431 21216962 PMC3048676

[13] Piskurich JF Lin KI Lin Y Wang Y Ting JPY Calame K. BLIMP-I mediates extinction of major histocompatibility class II transactivator expression in plasma cells. Nat Immunol. (2000) 1:526–32. 10.1038/82788 11101876

[14] Kim SJ Schatzle S Ahmed SS Haap W Jang SH Gregersen PK. Increased cathepsin S in Prdm1(-/-) dendritic cells alters the TFH cell repertoire and contributes to lupus. Nat Immunol. (2017) 18:1016–24. 10.1038/ni.3793 28692065 PMC5568473

[15] Goudot C Coillard A Villani AC Gueguen P Cros A Sarkizova S. Aryl hydrocarbon receptor controls monocyte differentiation into dendritic cells versus macrophages. Immunity. (2017) 47:582. 10.1016/j.immuni.2017.08.016 28930664

[16] Yu J Angelin-Duclos C Greenwood J Liao J Calame K. Transcriptional repression by blimp-1 (PRDI-BF1) involves recruitment of histone deacetylase. Mol Cell Biol. (2000) 20:2592–603. 10.1128/MCB.20.7.2592-2603.2000 10713181 PMC85475

[17] Su ST Ying HY Chiu YK Lin FR Chen MY Lin KI. Involvement of histone demethylase LSD1 in blimp-1-mediated gene repression during plasma cell differentiation. Mol Cell Biol. (2009) 29:1421–31. 10.1128/MCB.01158-08 19124609 PMC2648243

[18] Ancelin K Lange UC Hajkova P Schneider R Bannister AJ Kouzarides T. Blimp1 associates with Prmt5 and directs histone arginine methylation in mouse germ cells. Nat Cell Biol. (2006) 8:623–30. 10.1038/ncb1413 16699504

[19] Ren B Chee KJ Kim TH Maniatis T. PRDI-BF1/Blimp-1 repression is mediated by corepressors of the groucho family of proteins. Genes Dev. (1999) 13:125–37. 10.1101/gad.13.1.125 9887105 PMC316372

[20] Roberts NA Adams BD McCarthy NI Tooze RM Parnell SM Anderson G. Prdm1 regulates thymic epithelial function to prevent autoimmunity. J Immunol. (2017) 199:1250–60. 10.4049/jimmunol.1600941 28701508 PMC5544928

[21] Martins GA Cimmino L Shapiro-Shelef M Szabolcs M Herron A Magnusdottir E. Transcriptional repressor blimp-1 regulates T cell homeostasis and function. Nat Immunol. (2006) 7:457–65. 10.1038/ni1320 16565721

[22] Kallies A Hawkins ED Belz GT Metcalf D Hommel M Corcoran LM. Transcriptional repressor blimp-1 is essential for T cell homeostasis and self-tolerance. Nat Immunol. (2006) 7:466–74. 10.1038/ni1321 16565720

[23] Cheong C Matos I Choi JH Dandamudi DB Shrestha E Longhi MP. Microbial stimulation fully differentiates monocytes to DC-SIGN/CD209(+) dendritic cells for immune T cell areas. Cell. (2010) 143:416–29. 10.1016/j.cell.2010.09.039 21029863 PMC3150728

[24] Jang SH Chen H Gregersen PK Diamond B Kim SJ. Kruppel-like factor4 regulates PRDM1 expression through binding to an autoimmune risk allele. JCI Insight. (2017) 2:e89569. 10.1172/jci.insight.89569 28097234 PMC5214232

[25] Melnikov SV Manakongtreecheep K Rivera KD Makarenko A Pappin DJ Soll D. Muller's ratchet and ribosome degeneration in the obligate intracellular parasites microsporidia. Int J Mol Sci. (2018) 19:4125. 10.20944/preprints201811.0508.v1 30572624 PMC6321566

[26] Minnich M Tagoh H Bonelt P Axelsson E Fischer M Cebolla B. Multifunctional role of the transcription factor blimp-1 in coordinating plasma cell differentiation. Nat Immunol. (2016) 17:331–43. 10.1038/ni.3349 26779602 PMC5790184

[27] Fredriksson S Gullberg M Jarvius J Olsson C Pietras K Gustafsdottir SM. Protein detection using proximity-dependent DNA ligation assays. Nat Biotechnol. (2002) 20:473–7. 10.1038/nbt0502-473 11981560

[28] Soderberg O Gullberg M Jarvius M Ridderstrale K Leuchowius KJ Jarvius J. Direct observation of individual endogenous protein complexes in situ by proximity ligation. Nat Methods. (2006) 3:995–1000. 10.1038/nmeth947 17072308

[29] Kim SJ Goldstein J Dorso K Merad M Mayer L Crawford JM. Expression of blimp-1 in dendritic cells modulates the innate inflammatory response in dextran sodium sulfate-induced colitis. Mol Med. (2015) 20:707–19. 10.2119/molmed.2014.00231 25826676 PMC4398669

[30] Park Y Lee JM Hwang MY Son GH Geum D. Nono binds to the CpG island of oct4 promoter and functions as a transcriptional activator of oct4 gene expression. Mol Cells. (2013) 35:61–9. 10.1007/s10059-013-2273-1 23212346 PMC3887857

[31] Yadav SP Hao H Yang H-J Kautzmann M-AI Brooks M Nellissery J. The transcription-splicing protein NonO/p54^nrb^ and three NonO-interacting proteins bind to distal enhancer region and augment rhodopsin expression. Hum Mol Genet. (2014) 23:2132–44. 10.1093/hmg/ddt609 24301678 PMC3959818

[32] Zhang C Zhang MX Shen YH Burks JK Zhang Y Wang J. TNF-alpha suppresses prolyl-4-hydroxylase alpha 1 expression via the ASK1-JNK-NonO pathway. Arterioscl Throm Vas. (2007) 27:1760–67. 10.1161/ATVBAHA.107.144881 17478756 PMC2597036

[33] Lord CA Savitsky D Sitcheran R Calame K Wright JR Ting JPY. Blimp-1/PRDM1 mediates transcriptional suppression of the NLR Gene NLRP12/Monarch-1. J Immunol. (2009) 182:2948–58. 10.4049/jimmunol.0801692 19234190 PMC2701146

[34] Hatzi K Nance JP Kroenke MA Bothwell M Haddad EK Melnick A. BCL6 orchestrates Tfh cell differentiation via multiple distinct mechanisms. J Exp Med. (2015) 212:539–53. 10.1084/jem.20141380 25824819 PMC4387288

[35] Chan YH Chiang MF Tsai YC Su ST Chen MH Hou MS. Absence of the transcriptional repressor blimp-1 in hematopoietic lineages reveals its role in dendritic cell homeostatic development and function. J Immunol. (2009) 183:7039–46. 10.4049/jimmunol.0901543 19915049

[36] Chang DH Angelin-Duclos C Calame K. BLIMP-1: trigger for differentiation of myeloid lineage. Nat Immunol. (2000) 1:169–76. 10.1038/77861 11248811

[37] Yang R Masters AR Fortner KA Champagne DP Yanguas-Casas N Silberger DJ. IL-6 promotes the differentiation of a subset of naive CD8(+) T cells into IL-21-producing B helper CD8(+) T cells. J Exp Med. (2016) 213:2281–91. 10.1084/jem.20160417 27670591 PMC5068236

[38] Diehl SA Schmidlin H Nagasawa M Blom B Spits H. IL-6 triggers IL-21 production by human CD4(+) T cells to drive STAT3-dependent plasma cell differentiation in B cells. Immunol Cell Biol. (2012) 90:802–11. 10.1038/icb.2012.17 22491065 PMC3396759

[39] Nish SA Schenten D Wunderlich FT Pope S Gao Y Hoshi N. T cell-intrinsic role of IL-6 signaling in primary and memory responses. Elife. (2014) 3:e01949. 10.7554/eLife.01949 24842874 PMC4046568

[40] Li W Karwacki-Neisius V Ma C Tan L Shi Y Wu F. Nono deficiency compromises TET1 chromatin association and impedes neuronal differentiation of mouse embryonic stem cells. Nucleic Acids Res. (2020) 48:4827–38. 10.1093/nar/gkaa213 32286661 PMC7229820

[41] Hallier M Tavitian A Moreau-Gachelin F. The transcription factor Spi-1/PU.1 binds RNA and interferes with the RNA-binding protein p54nrb. J Biol Chem. (1996) 271:11177–81. 10.1074/jbc.271.19.11177 8626664

[42] Carotta S Willis SN Hasbold J Inouye M Pang SH Emslie D. The transcription factors IRF8 and PU.1 negatively regulate plasma cell differentiation. J Exp Med. (2014) 211:2169–81. 10.1084/jem.20140425 25288399 PMC4203955

[43] Nagy M Chapuis B Matthes T. Expression of transcription factors Pu.1, Spi-B, Blimp-1, BSAP and oct-2 in normal human plasma cells and in multiple myeloma cells. Br J Haematol. (2002) 116:429–35. 10.1046/j.1365-2141.2002.03271.x 11841448

