# Supplementary figures and images for: NonO Is a Novel Co-factor of PRDM1 and Regulates Inflammatory Response in Monocyte Derived-Dendritic Cells
Source: Front Immunol. 2020 Jul 10;11:1436. doi: 10.3389/fimmu.2020.01436 (PMC7378894; doi:10.3389/fimmu.2020.01436)

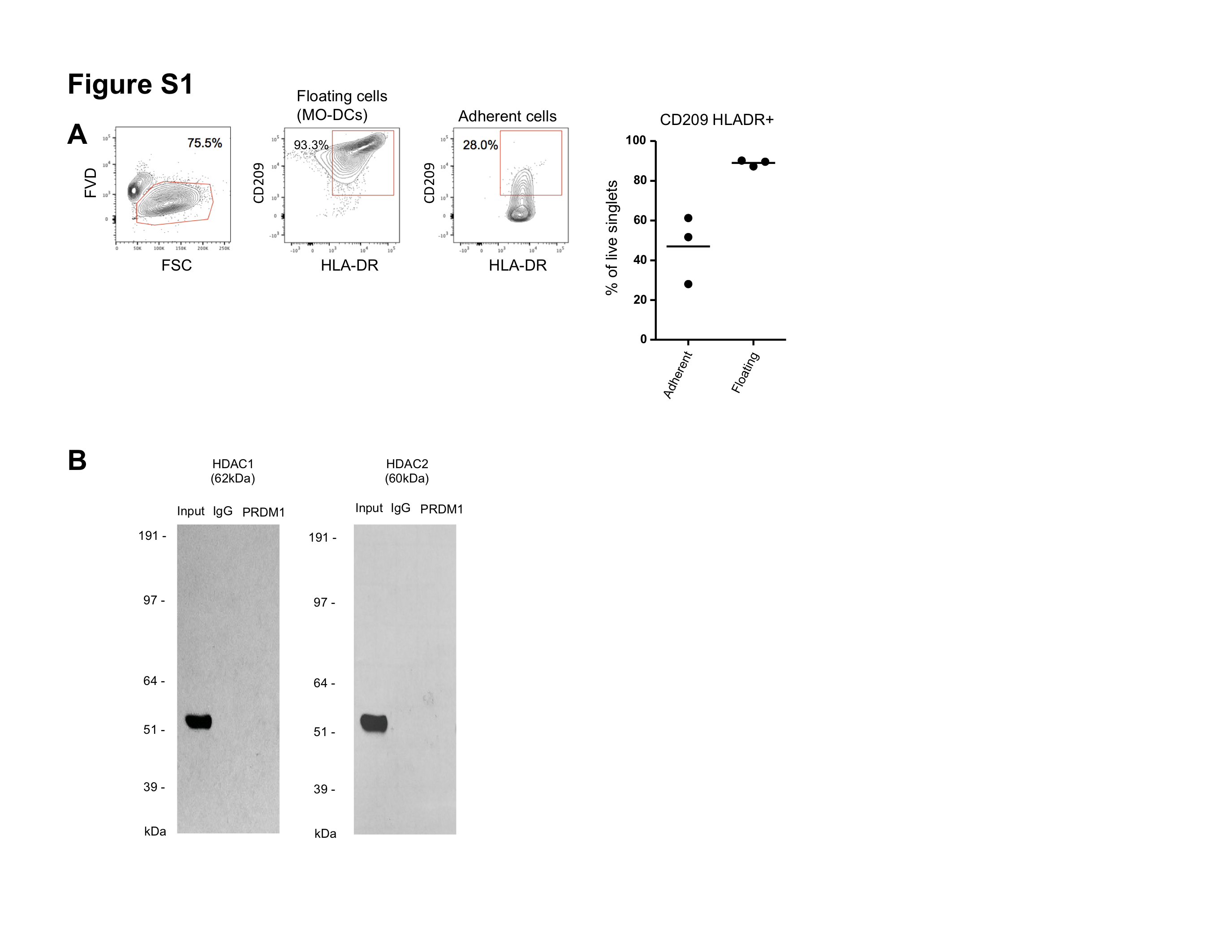

Supplement: Figure S1 — (A) A representative flow image of MO-DCs. Day 7 of MO-DCs (non-adherent cells) and adherent cells were collected and viability and the purity were investigated by exclusion of live/dead fixable marker, FVD and HLA-DR and CD209 antibodies. Purity of MO-DC was calculated by percentage of live HLA-DR/CD209 double positivity. (B) Assessment of HDAC binding to PRDM1 by Co-IP. Nuclear fraction of MO-DCs was immunoprecipitated with anti- PRDM1 antibodies or control IgG and immunoblotting was performed with anti-HDAC1 or anti-HDAC2 antibodies. A representative image from two independent experiments is shown. [file Image_1.TIFF]

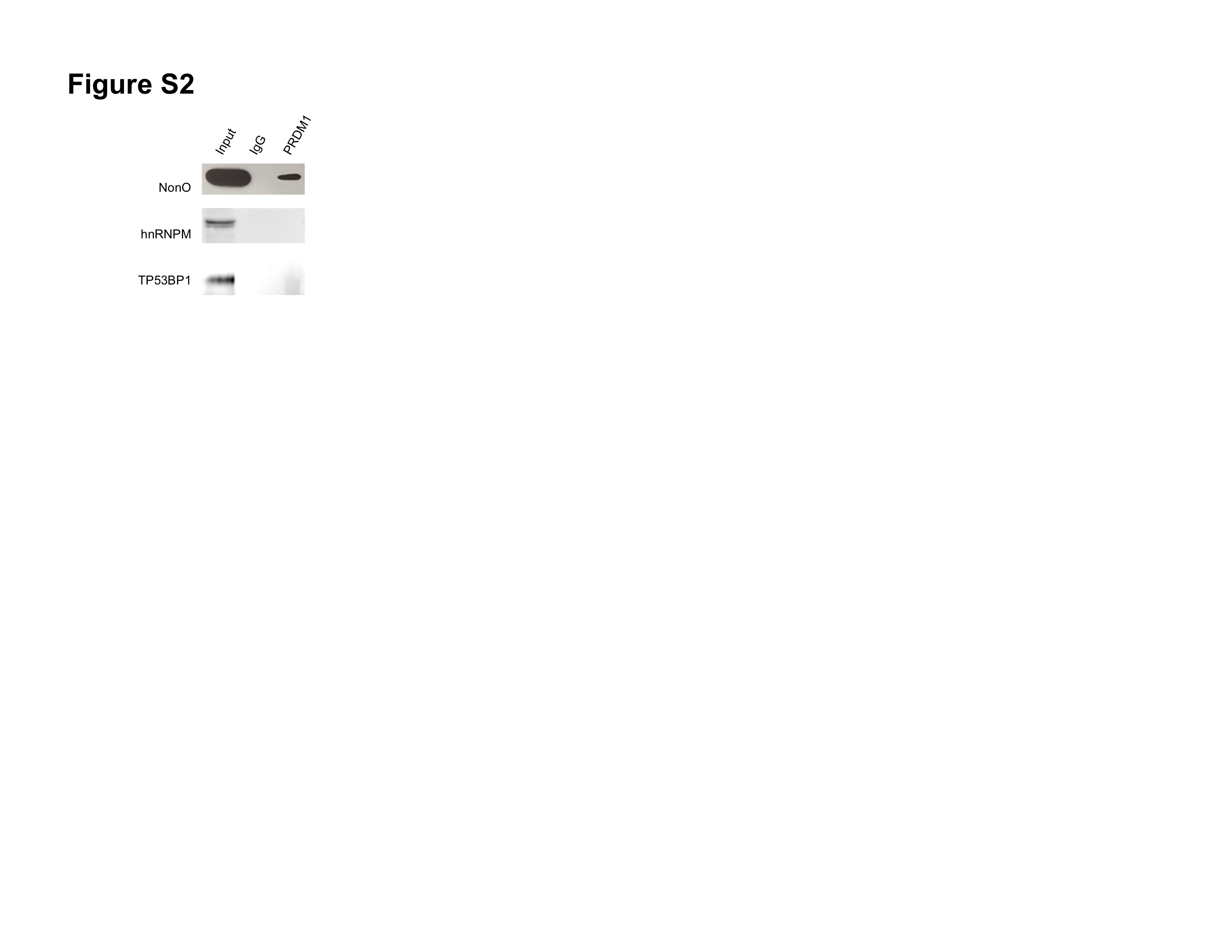

Supplement: Figure S2 — Binding of PRDM1 with NonO, hnRNPM, and TP53BP1 by Co-IP. Nuclear fraction of MO-DCs were incubated with ant-PRDM1 or control IgG and immunoblotting was performed with anti-NonO, hnRNPM, or TP53BP1 antibodies. Input is an unfractionated total nuclear extract. A representative image of two independent experiments. [file Image_2.TIFF]

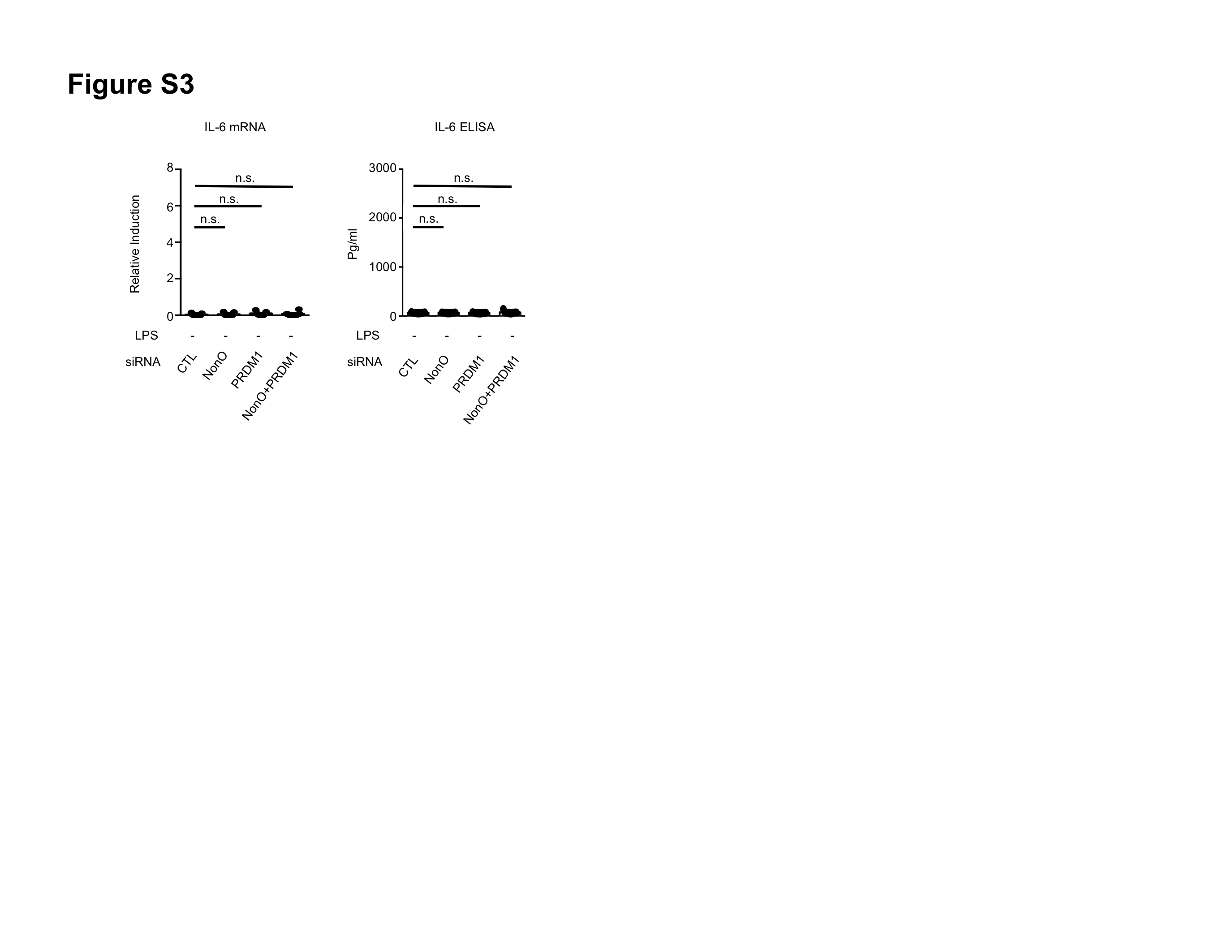

Supplement: Figure S3 — The basal level of IL-6 transcript and protein were measured by qRT-PCR and ELISA. NonO, PRDM1, and both (NonO and PRDM1) siRNA or control siRNA transfected MO-DCs were cultured without LPS (1 μg/ml) for 6 h, and total RNA was purified. Relative level of IL-6 was measured by qRT-PCR and normalized to the level of housekeeping gene, POLR2A. Supernatant concentrations of IL-6 obtained from the cultures were measured using ELISA. Bar graph is a mean ± SEM (n = 9). Significance determined by Mann Whitney test. [file Image_3.TIFF]

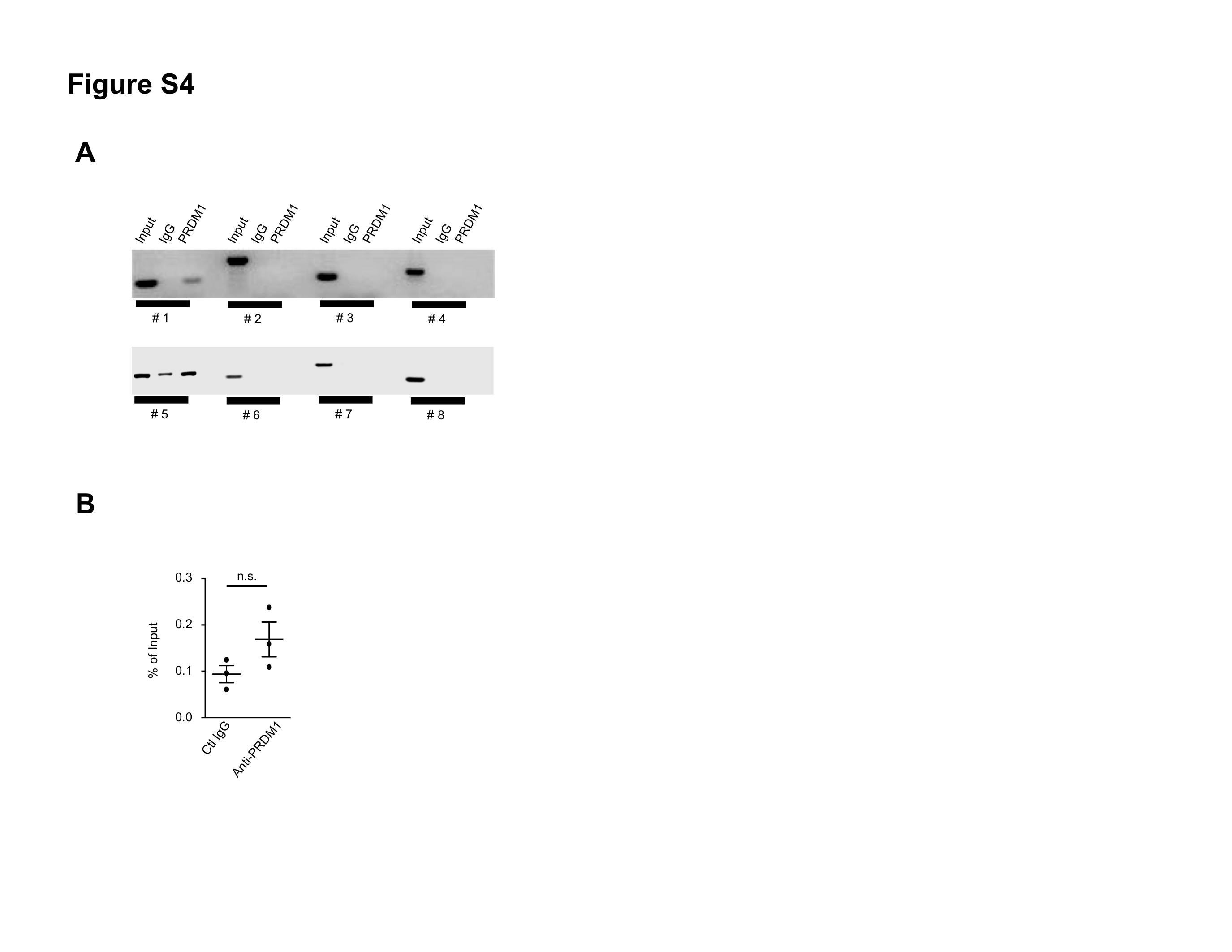

Supplement: Figure S4 — Assessment of PRDM1 binding to IL6 promoter regions by ChIP-qPCR. To test PRDM1 binding to IL6 promoter, ChIP was performed. Nuclear fraction of MO-DCs and ChIP was performed by anti-RPDM1 or control IgG as described in material and method. PCR (A) or qPCR (B) was performed to assess binding of PRDM1 by primers described in material methods. #1–#8 indicates each region including putative PRDM1 binding sites in IL6 promoter. (A) is a representative image of three independent experiments. (B) To quantify the binding of PRDM1 to #5 region, qPCR was performed and calculated by the percent of input. Each dot represents an individual sample and the bar represents the mean ±SEM (n = 3). Significance determined by Mann Whitney test. [file Image_4.TIFF]

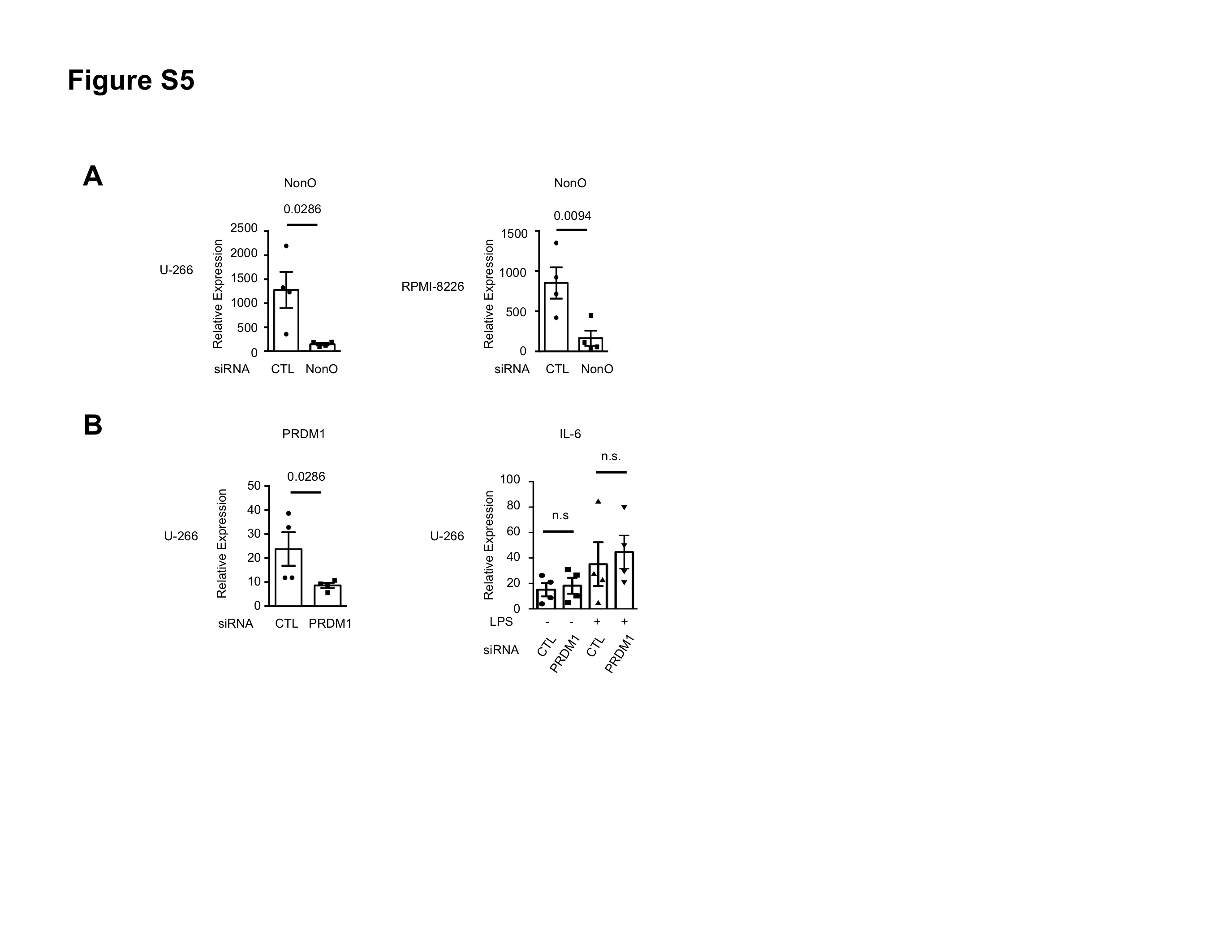

Supplement: Figure S5 — Expression of IL6 by NonO or PRDM1 in myeloma cells. (A) NonO expression was knock down by transfection of anti-NonO siRNA or scrambled control siRNA. After transfection, relative level of NonO was measured by qRT-PCR and normalized to the level of housekeeping gene, POLR2A. (B) To knock down the PRDM1 expression, anti-PRDM1 siRNA, or control siRNA was transfected to U266 cells and PRDM1 level was measured by qRT-PCR. U266 cells transfected with control or anti-PRDM1 siRNA was cultured with or without LPS (40 μg/ml) for 6 h. Relative level of PRDM1, IL6 was normalized to the level of POLR2A. Each dot represents an individual sample and the bar is the mean ± SEM. Significance determined by Mann Whitney test. [file Image_5.TIFF]

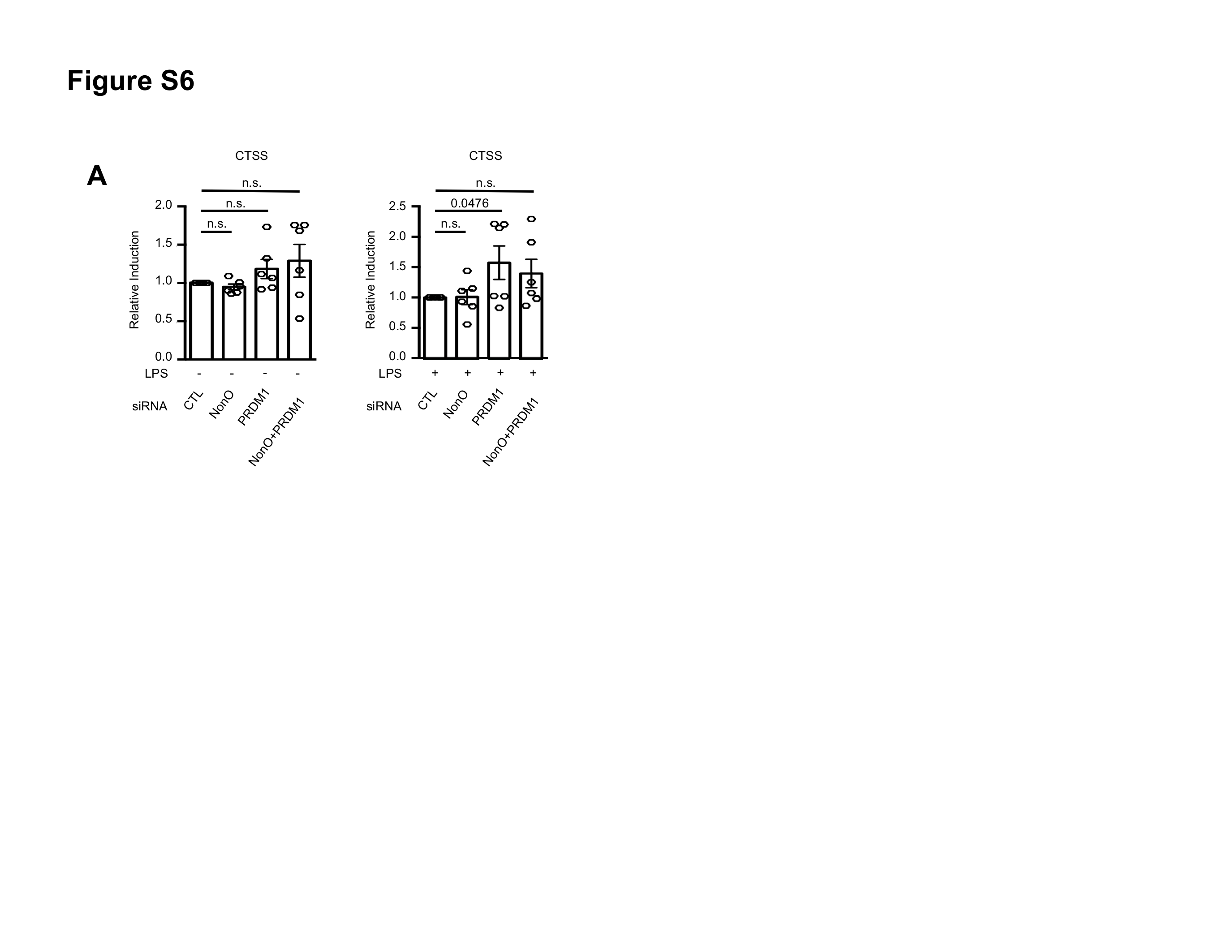

Supplement: Figure S6 — The level of CTSS was measured by qRT-PCR. NonO, PRDM1, and both (NonO and PRDM1) siRNA or control siRNA transfected MO-DCs were cultured with or without LPS (1 μg/ml) for 6 h, and total RNA was purified. Relative level of CTSS was measured by qRT-PCR and normalized to the level of housekeeping gene, RPLP0. Then, relative induction was calculated by normalization to the level of control. Bar graph is a mean ± SEM (n = 6). Significance determined by Mann Whitney test. [file Image_6.TIFF]

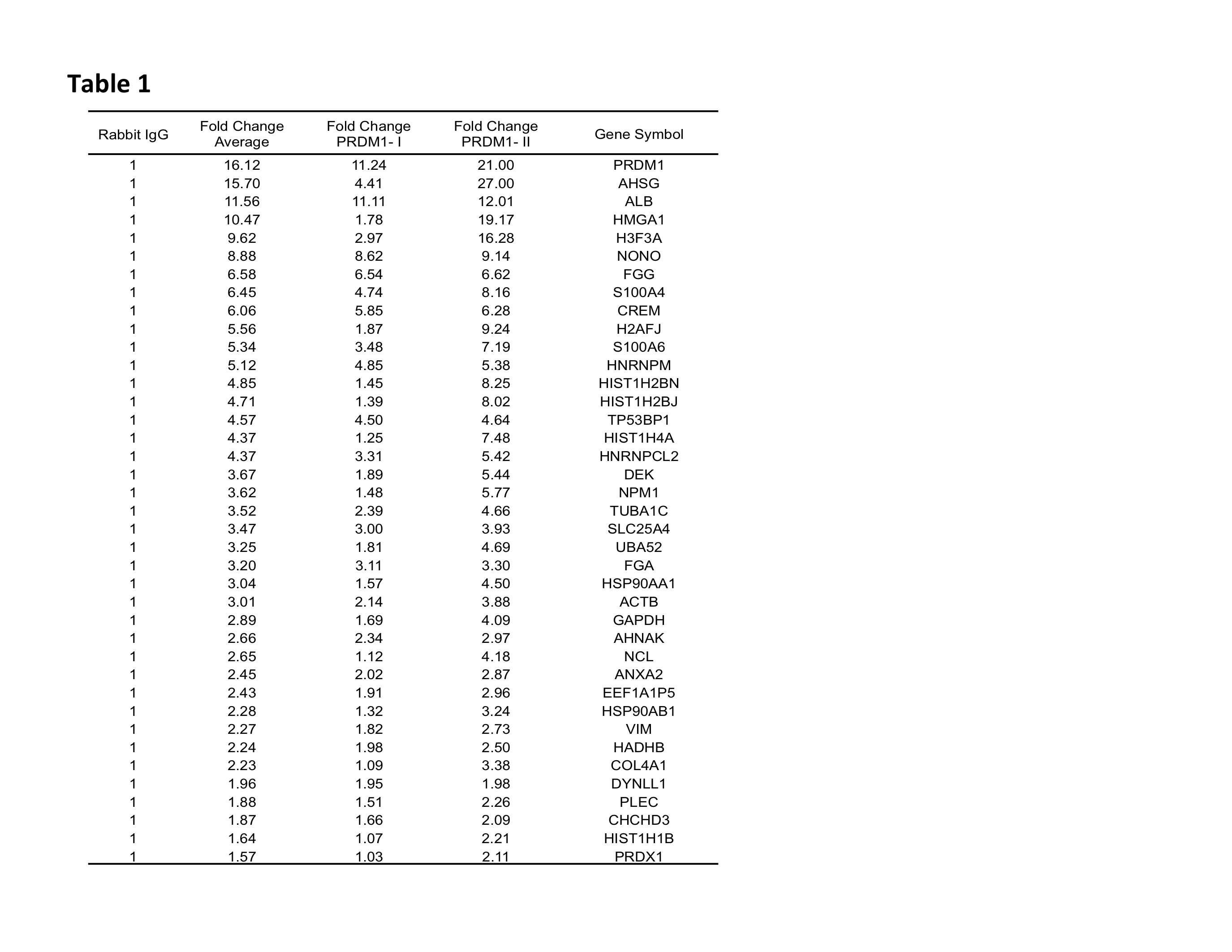

Supplement: Table S1 — Mass spectrometric identification of candidate PRDM1 binding proteins in MO-DCs. The comparative analysis of peptide and protein quantification in normal IgG and PRDM1 of PRDM1-sufficient MO-DCs are subjected through iTRAQ-based quantitative proteomics with cutoff >1.5-fold. The experiment was repeated two times. iTRAQ, isobaric tags for relative and absolute quantitation; MO-DCs, monocyte derived-dendritic cells. [file Image_7.TIFF]
